# Engraftment of aging-related human gut microbiota and the effect of a seven-species consortium in a pre-clinical model

**DOI:** 10.1080/19490976.2023.2282796

**Published:** 2023-11-27

**Authors:** Huimin Ye, Tarini S. Ghosh, Cara M. Hueston, Klara Vlckova, Anna V. Golubeva, Niall P. Hyland, Paul W. O’Toole

**Affiliations:** a School of Microbiology, University College Cork, Cork, Ireland; b APC Microbiome Ireland, University College Cork, Cork, Ireland; c Department of Anatomy and Neuroscience, University College Cork, Cork, Ireland; d Department of Physiology, University College Cork, Cork, Ireland

**Keywords:** Microbiota, elderly, germ-free, murine, aging, inflammation, frailty

## Abstract

Human aging is characterized by gut microbiome alteration and differential loss of gut commensal species associated with the onset of frailty. The administration of cultured commensal strains to replenish lost taxa could potentially promote healthy aging. To investigate the interaction of whole microbiomes and administered strains, we transplanted gut microbiota from a frail or healthy elderly subject into germ-free mice. We supplemented the frail-donor recipient group with a defined consortium of taxa (the “S7”) that we identified by analyzing healthy aging subjects in our previous studies and whose abundance correlated with health-promoting dietary intervention. Inoculation with a frail or a healthy donor microbiome resulted in differential microbiota compositions in murine recipients 5 weeks post-transplantation. Fecal acetate levels were significantly higher in healthy donor recipient mice than in frail donor recipient mice after 4 weeks. However, the frailty-related phenotype was not replicated in recipient mice with single-dose microbiota transplantation from a healthy and a frail donor. Five S7 species colonized successfully in germ-free mice, with a relatively high abundance of *Barnesiella intestinihominis* and *Eubacterium rectale*. The engraftment of five S7 species in germ-free mice increased fecal acetate levels and reduced colon permeability and plasma TNF-ɑ concentration. Supplementation with the S7 in frail-microbiota recipient mice did not increase alpha-diversity but significantly increased the abundance of *Barnesiella intestinihominis*. S7 supplementation showed the potential for improving spatial reference memory in frail-microbiota recipient mice. Collectively, these data highlight the challenge of elderly microbiota engraftment in the germ-free mouse model but show promise for modulating the gut microbiome of frail elderly subjects by administering an artificial gut microbe consortium associated with healthy aging.

## Introduction

Aging is defined as the time-dependent functional decline that increases vulnerability to death,^1^ and maintaining functional ability is considered “healthy” aging. However, older people (typically 65 y old and above) experience reduced function of multiple systems, including energy harvest, physical ability, and cognition, collectively contributing to different extents of frailty. When frailty becomes established, functional decline is accelerated and homeostatic mechanisms start failing.^2^ Research and practice seeking to promote healthy aging aims to delay the onset of frailty or even avoid developing chronic diseases.

Aging is linked to gut microbiota alteration associated with chronic inflammation, gut permeability changes, and physical and cognitive dysfunction.^3^ The microbiota of older people is characterized by lower alpha diversity, reduced abundance of subdominant taxa, and the loss of bifidobacterial and fiber-responsive taxa compared to that of younger adults.^4,5^ An increase in the proportional abundance of pathobionts has also been reported in people above 100 y old.^6–10^ Aging-related microbiota changes can potentially make the host more susceptible to certain diseases, accelerating functional decline in the frail elderly. We previously identified a group of multiple disease-associated taxa in elderly subjects that differ from those involved in microbiome alterations in non-communicable diseases in younger people.^11^ In general, the gut microbiota of the elderly with higher frailty is characterized by loss of commensals associated with health at all ages and gain of disease-associated pathobionts that also relate to medication, reduced mobility, and consumption of a less diverse diet.^3^

Diet-based and microbe-based interventions are thus the two main strategies for microbiome modulation in the elderly. A previous study from our laboratory has shown that fiber-associated taxa were difficult to restore by administration of a mix of five prebiotics for 6 months in the elderly.^12^ Microbe-based strategies include fecal microbiota transplantation (FMT) and live biotherapeutics. FMT is a valid clinical option for treating *Clostridioides difficile* infections (CDI), but there are operational challenges, such as occasional adverse events and the requirement for strict donor screening.^13–15^ Administering a defined consortia of cultured bacteria is an alternative therapeutic strategy to fecal transplants. It has been shown that a synthetic bacterial suspension consisting of 13 microbial species is an efficacious therapeutic for CDI.^16^ Current probiotic supplement studies are based on recognized probiotics, usually *Lactobacillus* or *Bifidobacterium* species.^17–20^ Despite the reported high efficacy of those probiotics in infants, children, and young adults,^20,21^ the associations of these species with healthy aging have not been clearly demonstrated in the elderly. In contrast to younger people who gradually gain disease-associated gut microbes, people older than 50 tend to lose gut commensals.^22^ Thus, microbiome-based therapeutic strategies targeting the elderly could attempt to restore selected commensals in a consortium format to prevent or delay the onset of frailty and age-related inflammation

The importance of some specific species in the aging-subject microbiome and the potential for therapeutic use of defined bacterial consortia has gathered momentum as the role of microbiota in healthy aging is elucidated. Characteristic changes in the microbiota composition of older people are correlated with frailty, co-morbidity, and inflammation markers despite the great inter-individual variation of the microbiota of older people (aged 60 and above).^11,23^ Previous diet intervention studies have revealed that specific taxa whose abundances are increased by a Mediterranean diet are positively associated with lower frailty and improved cognitive function in older people over 65.^24^ A previous study reported significantly different abundances of 17 gut microbes between “highly frail” and “low frail” individuals aged over 70.^25^ A repertoire of frailty-related genus-level taxa was identified by us in higher abundance in Long-stay subjects compared to Community-dwelling subjects of the ELDERMET cohort (aged above 60).^3^ A distinctive microbiota configuration associated with long-term care and frailty has also been identified.^26^ In a recent study, we extensively cultured taxa above 1% relative abundance in healthy community-dwelling subjects and used the ensuing Microbiome Culture Collection 100 (MCC100) strains to modulate the gut microbiota of elderly donors in an *in vitro* human colon model.^27^ Supplementation of the artificial colon with the MCC100 increased microbiome alpha-diversity and the levels of health-associated amino acids, motivating us to develop and refine this approach.

Compositional or functional changes in gut microbiota have been associated with various diseases, including inflammatory bowel disease,^28^ colorectal cancer,^29,30^ and metabolic syndrome.^31^ However, establishing causal relationships between microbes and disease conditions is complicated by highly individualized microbiomes and regulatory issues for administering live biotherapeutics in human intervention studies. Human microbiota-associated (HMA) mice are a powerful model for studying the involvement of gut microbiota in human health. The “Humanization” process involves the transplantation of human gut microbiota into germ-free (GF) mice to establish human gut microbial communities in mouse models. Many human fecal microbiota transplantation studies have successfully recapitulated human donor phenotypes such as obesity,^32^ allergies,^33^ inflammatory bowel disease,^34^ and autism.^35^ HMA mice hold promise for studying the association of gut microbiota with frailty in the elderly. However, the extent to which the recipient microbiota represents that of the donor following transplant is not always assessed. It is unknown whether the transplantation of elderly human microbiota to GF mice could recapitulate the human donor phenotype, in this case, frailty.

To establish a preclinical humanized mouse model for aging studies, we first identified a healthy aging-associated bacterial consortium – the S7 species. We then conducted transplantation of gut microbiota from a frail and a healthy elderly subject to GF mice to replicate human frailty phenotypes. We also investigated the potential of the S7 consortium for counteracting gut permeability changes, physical and cognitive decline, and inflammation.

## Results

**Figure 1.**
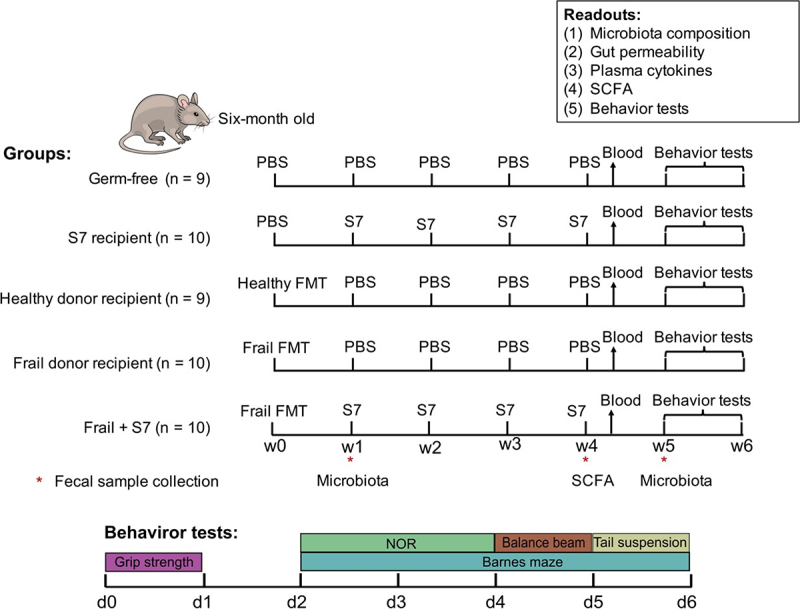
Overview of the experimental design and study timeline.

Despite the highly individualized microbiota of elderly subjects, many taxa have been reported to be associated with frailty. Thus, this study aimed to investigate whether the engraftment of the microbiota of donors with different levels of frailty, namely a “frail” donor and a “healthy” donor, could replicate human frailty-related phenotypes using a germ-free mouse model. Furthermore, the effect of administering a seven-species consortium (the S7) linked to healthy aging was examined (Figure 1).

Germ-free mice were inoculated with the gut microbiota of frail elderly subjects and optionally supplemented with seven selected strains from the MCC100 collection (S7). Control groups consisted of germ-free uninoculated, mice inoculated S7 only, frail donor recipient control inoculated with the gut microbiota of a frail human subject, and healthy donor recipient control inoculated with a healthy human microbiota sample. Vertical bars represent one-week/day intervals. Behavior tests conducted included balance beam, novel object recognition (NOR), grip strength, tail suspension, and Barnes maze. Blood samples were collected from the tail to measure proinflammatory cytokines and intestinal barrier function (*in vivo* FITC measurement). Asterisks indicate the fecal sample collection. Fecal samples collected at weeks 1 and 5 were used for 16S rRNA gene amplicon sequencing. Fecal samples obtained at week 4 were used for short-chain fatty acids measurement. Colon tissues were collected after sacrifice and used for *ex-vivo* FITC measurement.

### Engraftment and temporal abundance trajectory of elderly human gut microbiota in germ-free mice

Representative “healthy” donor and “frail” donor (*n* = 1) was selected based upon their clinical and health measurement and microbiome relatedness to a subset of the well-studied ELDERMET cohort (Supplementary Figure S1a, Supplementary Table S1). Our sequencing data showed that microbial compositions of frozen donor samples are similar to corresponding fresh fecal samples with a 0.03 Jensen-Shannon Divergence (JSD)^36^ between fresh and frozen healthy donor samples and a 0.04 JSD between fresh and frozen frail donor samples. Furthermore, all taxa in fresh fecal samples can be detected in frozen fecal samples at family level (Supplementary Figure S1 a and b). The plating results showed that frail and healthy donor samples remained 10^9^ CFU/ml after storage at −80°C, suggesting that the sample storage did not affect the microbiota of selected donors. Most importantly, the 16S rRNA sequencing results confirmed that the typical community or long-stay microbiota profiles were present in the donors.

**Figure 2.**
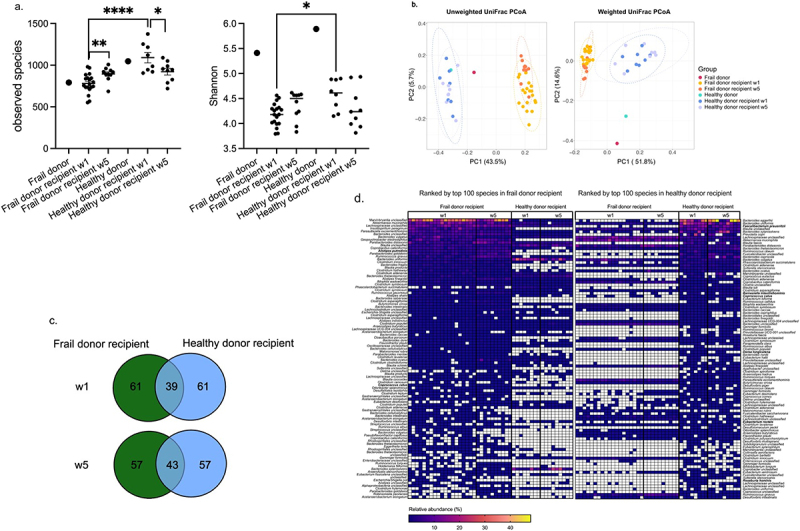
Engraftment of human donor microbiota in murine recipients.

The efficacy of taxon transfer from frozen elderly donor fecal microbiota was evaluated in germ-free (GF) mice using a single healthy or frail donor. GF mice were humanized with single oral gavage of healthy or frail microbiota at week 0 (as shown schematically in Figure 1). The total bacterial numbers in healthy and frail donor feces were measured by qPCR as 5.0 × 10^9^ and 3.4 × 10^9^ genome copies/g, respectively. The gut microbiota of the healthy donor showed higher alpha-diversity indices (numbers of observed species and Shannon Index) than the frail donor microbiota (Figure 2a). As expected, the frail and healthy donor stools harbored different microbiota compositions, with a higher relative abundance of *Firmicutes* and *Proteobacteria* and a lower relative abundance of *Bacteriodota*, *Actinobacteriota*, and *Desulfobacterota* in the frail donor compared with the healthy donor (Supplementary Figure S2a).

Fecal samples of recipient mice were collected at 1 and 5 weeks post-FMT for 16S rRNA gene amplicon sequencing. **a**. Alpha-diversity was measured using Observed Species and Shannon Index. Data were expressed as Mean ± SEM. One-way ANOVA post hoc Holm-Sidak, * *P* <.05, ** *P* <.01, **** *P* <.0001. **b**. Principal coordinates analysis (PCoA) plots based on unweighted and weighted UniFrac distances. Each dot represents one individual (human donor or mouse). **c**. Venn diagram shows the shared species number in the top 100 species of frail and healthy donor recipient mice. **d**. The composition of the top 100 species of frail donor recipient mice differs from that of healthy donor recipient mice at weeks 1 and 5. The top 100 species were selected by ranking the mean relative abundance of the species from frail and healthy donor recipient mice at week 1. S7 species are in bold, and species with <0.01% relative abundance are colored white. Each column represents an individual. *n* = 19 (Frail donor recipient w1), *n* = 10 (Frail donor recipient w5), *n* = 8 (Healthy donor recipient w1), *n* = 9 (Healthy donor recipient w5).

Fecal samples were collected from recipient mice for microbiome profiling by 16S rRNA gene amplicon sequencing at weeks 1 and 5 post-transplantation. Measurement of the alpha-diversity revealed significantly lower numbers of observed species (*p* <.0001, One-way ANOVA post hoc Holm-Sidak) and Shannon index (*p* =.016, One-way ANOVA post hoc Holm-Sidak) values in frail donor recipient mice compared with healthy donor recipient mice at 1-week post-transplantation (Figure 2a). Unweighted and weighted UniFrac distances analysis revealed distinct frail and healthy donor microbiota in the germ-free mouse model 1 week after the transplantation. The differences remained up to 5 weeks post-transplantation (Figure 2b), whereas no differences were observed in alpha diversity between healthy and frail donor recipient mice at week 5. Pairwise similarity analysis revealed significant differences in Unweighted (*p* =.001) and Weighted (*p* =.001) UniFrac distances between the microbiota of frail donor recipient mice at weeks 1 and 5. Notably, there was no significant difference in weighted UniFrac distances between healthy donor recipient mice at weeks 1 and 5 (*p* =.13), suggesting more stable colonization of healthy elderly microbiota in germ-free mice than frail elderly microbiota (Supplementary Table S2).

16S rRNA profiling of fecal samples obtained from frail donor recipient mice showed partial engraftment of the donor gut microbiota: 7/9 phyla colonized at both weeks 1 and 5, while only 51% (47/93) at week 1 and 54% at week 5 (50/93) of genus-level taxa of the frail donor were detected among the recipient mice (Supplementary Table S3 and S4). In contrast, 7/8 phyla from healthy donor stool colonized at both week 1 and week 5, 66% (76/115) and 65% (75/115) of healthy donor genus-level taxa were detected in the recipient mice at weeks 1 and 5, respectively.

Evaluation of family-level microbial community composition in recipient mice revealed a greater relative abundance of the families *Bacteroidaceae* and *Akkermansiaceae* in mouse communities compared to humans, indicating the adaptation of those human bacteria in the murine gut environment. Additionally, *Rikenellaceae*, *Tannerellaceae*, *Sutterellaceae*, and *Erysipelatoclostridiaceae* exhibited higher relative abundance in the frail donor recipient mouse gut than that of the frail human donor itself, which was not observed in healthy donor recipients (Supplementary Figure S2b). We then performed a more refined analysis of the engraftment capacity of donor microbiota. For this purpose, we computed pairwise Spearman correlations between recipient mice and their donor using the relative abundances in each fecal sample at the genus/family level. Correlation analysis revealed higher similarity (*p* <.0001, One-way ANOVA post hoc Holm-Sidak) between healthy donor recipient mice and healthy donor (genus 0.46 ± 0.03, family 0.65 ± 0.01) compared to the similarity between frail donor recipient and frail donor (genus 0.27 ± 0.01, family 0.53 ± 0.01) at week 1. Similarly, the engraftment efficacy of healthy donor microbiota was greater than that of frail donor microbiota at week 5 (Supplementary Table S5, Supplementary Figure S3).

We next compared the top 100 species from the frail donor and healthy donor with their mouse recipients to assess species engraftment. The presence of the species in >50% of the recipient samples was considered successful engraftment, and <10% was considered failed engraftment. Notably, more species from the healthy donor were detected in the respective recipient murine gut compared with the frail donor species (Supplementary Figure S2c). In the case of frail donor microbiota, 38% of the top 100 species engrafted successfully in mouse recipients, while 33% of the top 100 species could not be recovered from the murine gut. In contrast, 60% of the healthy donor microbiota species engrafted successfully, and only 25% of the top 100 species failed to colonize the mouse gut. The engraftment efficacy reviewed at different taxonomic levels revealed that the healthy donor microbiota engrafted more effectively in the GF mouse model than the frail donor microbiota.

To compare the gut microbiota composition of frail and healthy donor recipient mice, we identified the top 100 species from the recipient animal microbiota. Frail and healthy donor recipient mice shared only 39 and 43 of the top 100 species at weeks 1 and 5, respectively (Figure 2c). Interestingly, some of the most abundant species in frail donor recipient mice were present with low abundance in healthy donor recipient mice, such as *Bacteroides cellulosilyticus* (24% vs. 0.04%), *Akkermansia muciniphila* (7% vs. 1.7%), and *Lachnospiraceae* unclassified (9% vs. 2.6%). Likewise, highly abundant species in healthy donor recipients, including *Bacteroides eggerthii* (24% vs. 0.01%), *Bacteroides uniformis* (12% vs. 1.1%), and *Faecalibacterium prausnitzii* (7.6% vs. 0.005%), are present with low abundance in frail donor recipient mice (Figure 2d).

Collectively, these data revealed a greater engraftment efficacy of healthy elderly microbiota taxa than those of frail elderly microbiota. The engraftment of healthy or frail human microbiota resulted in distinct microbial composition in recipient mice, which were retained for up to 5 weeks post-transplantation.

### The relative abundances of the S7 consortium are negatively associated with frailty in the elderly

**Figure 3.**
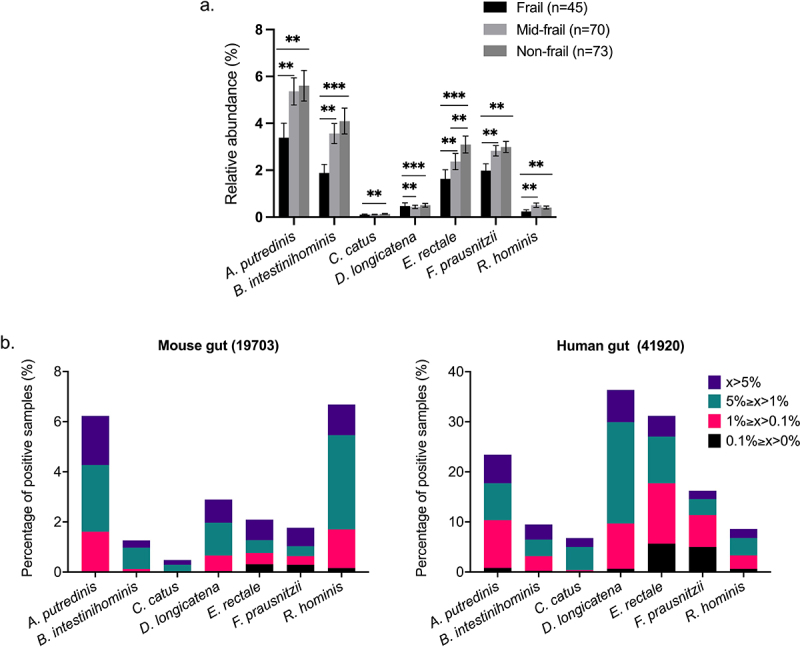
Prevalence and abundance of S7 taxa in human and mouse gut microbiome datasets.

By analyzing disease- and diet-associated taxa in elderly subjects, a candidate group of species (that we refer to here as the S7), consisting of *Alistipes putredinis*, *Barnesiella intestinihominis*, *Coprococcus catus*, *Dorea longicatena*, *Eubacterium rectale*, *Faecalibacterium prausnitzii*, and *Roseburia hominis*, was identified as frailty-associated taxa (see Methods). To verify the correlation between these selected taxa and frailty, we re-analyzed the shotgun fecal microbiome data of 188 elderly Irish individuals (64–102 y old) from the ELDERMET cohort (Supplementary Table S6). Our results showed that the abundances of *A. putredinis*, *B. intestinihominis*, *C. catus*, *D. longicatena*, *E. rectale*, *F. prausnitzii*, and *R. hominis* were differentially negatively associated with frailty (Figure 3a). In the ELDERMET cohort, *A. putredinis* is the most abundant species of the S7 species across all frailty categories, followed by *B. intestinihominis*, *E. rectale*, and *F. prausnitzii*. Relative abundances of *C. catus*, *D. longicatena*, and *R. hominis* are relatively low (<0.1%) in the selected Irish population (Supplementary Table S7). We previously isolated the Microbiome Culture Collection 100 (MCC100) strains from fecal samples of healthy donors.^27^ The genomes of the MCC100 strains had been sequenced (Supplementary Table S8), and their antimicrobial susceptibility was assessed.^27^ The availability of cultured isolates of the healthy aging-associated S7 species in our MCC100 collection facilitated their usage in the current study.

a. Differential abundance by fragility of S7 taxa in 188 ELDERMET subjects with available Functional Independence Measure (FIM) and Barthel Scores. Frail, FIM ≤ 90; Mid-frail, 90 < FIM < 126; Non-frail, FIM = 126. Bars represent the Mean ± SEM. Kruskal–Wallis post hoc Dunn's test, **P <.01, ***P <.001. b. Occurrence and prevalence analysis of S7-related sequences in human and mouse gut using the Integrated Microbial Next-Generation Sequencing (IMNGS) platform. S7-like sequences at 99% similarity cutoff are expressed as percentages of positive samples in each host (the sample numbers used for the analysis are shown in parentheses), and different colors indicate the S7-related sequences at different relative abundance ranges.

There are very few published studies of fecal microbiota transplantation (FMT) from elderly human donors to GF mice, so we performed a meta-analysis to measure the presence and relative abundance of the S7-related sequences in the gut of human and mice using published 16S rRNA gene amplicon datasets contained in IMNGS.^37^ Although the prevalence of S7-related sequences (99% similarity) is lower in the mouse gut compared to the human gut samples, the presence of all seven species was detected in the mouse gut, suggesting the possibility of S7 colonization in the mouse gut. The observed patterns of higher prevalence of S7-related taxa in humans compared with mice support their being regarded as human gut bacteria (Figure 3b).

### Engraftment of the S7 species in germ-free and frail donor recipient mice

**Figure 4.**
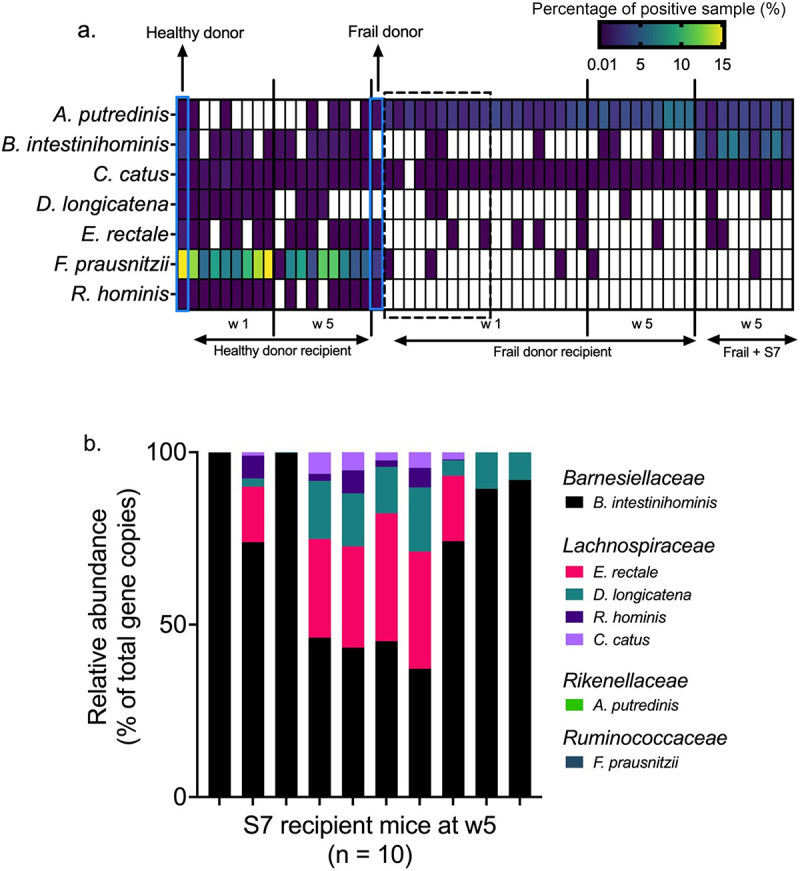
Effect of S7 supplementation on the microbiome of frail donor recipient mice.

We first assessed the engraftment of the S7 species originating from the human donor microbiota. We applied a threshold of presence in at least 50% of the recipient mice as successful engraftment. Seven and five S7 species were detected in healthy and frail donor microbiota, respectively. Six of seven S7 species originating from healthy donor microbiota were detected at week 1, and five were retained at week 5. Only two frail donor-originated S7 species were detected at weeks 1 and 5 (Figure 4a), suggesting different engraftment efficacies of S7 species in distinct microbial communities.

a. Distribution of S7 taxon in human donors and mouse recipients at weeks 1 and 5. Only taxa with >0.01% relative abundance are shown. Frail donor recipient mice at week 1, further treated with S7, are marked with dashed lines. *n* = 19 (Frail donor recipient w1), *n* = 10 (Frail donor recipient w5), *n* = 8 (Healthy donor recipient w1), *n* = 9 (Healthy donor recipient w5). b. Relative abundance of S7 taxa in the gut of S7 recipient mice after four inoculations of the S7 consortium. Each S7 species was quantified using S7 strain-specific primers. Each bar represents one animal (*n* = 10).

To investigate the engraftment of the defined S7 consortium, we supplemented GF (and no other microbial inoculum) and frail-donor recipient mice with the S7 consortium, as shown in Figure 2. Engraftment efficacy of S7 in GF mice was assessed using S7 strain-specific 16S rRNA primers. Five S7 species successfully colonized the S7-mono-associated GF mice, with the highest proportional abundances achieved by *B. intestinihominis*, followed by *E. rectale*, *D. longicatena*, *R. hominis*, *C. catus* (Figure 4b). Colonization of *A. putredinis* and *F. prausnitzii* was not detected in GF mice. Next, we examined the engraftment of the S7 in frail donor recipient mice to test if the treatment with the S7 consortium might attenuate the difference between the gut microbiota of frail- and healthy-donor recipient mice. While the supplementation with S7 did not change the microbiota alpha-diversity and weighted UniFrac beta-diversity of frail-donor recipient mice, unweighted UniFrac beta-diversity was affected by S7 treatment (*R*^*2*^ = .09, *p* =.007) (Supplementary Figure S4 a and b). At the species level, the S7 treatment primarily increased the relative abundance of *B. intestinihominis* in frail donor recipient mice (Figure 4a). DESeq2 analysis^38^ further revealed an increased abundance of *Bacteroides* and *Lachnospiraceae*, and a lower abundance of *Clostridium saccharogumia* in frail-donor recipient mice receiving the S7 supplementation (Supplementary Figure S4c). However, all taxa mentioned above, except *B. intestinihominis*, were present with low relative abundances (<0.2%).

### Effect of S7 consortium on fecal SCFA levels

Fecal samples were collected at week 4 for short-chain fatty acid (SCFA) measurement (as shown schematically in Figure 1). Healthy donor recipient mice displayed higher fecal acetate (*p* =.036, one-way ANOVA post hoc Holm-Sidak) levels compared to frail donor recipient mice 4 weeks post-transplantation (Supplementary Figure S5). We observed no differences in microbial metabolites between frail donor-recipient mice with or without S7 supplementation. However, S7 supplementation in germ-free mice increased acetate levels and decreased glucose concentrations (*p* <.001, one-way ANOVA post hoc Holm-Sidak) compared with the germ-free controls. Lactose, succinic acid, and butyric acid were not detected in all samples, and there was no difference in lactose and formic acid between groups.

### Inflammatory cytokine alteration before and after restraint stress

Transplantation of gut microbiota from old mice (17 months) to young mice (7–10 weeks) has been shown to affect inflammaging.^39^ Proinflammatory cytokines, such as TNF-ɑ, IL-6, and MCP-1, are considered relevant mediators during the onset of frailty.^40–42^ Thus, we examined aging-related inflammatory cytokines in plasma, specifically TNF-ɑ, IL-6, and MCP-1. We tested the plasma cytokine concentrations before and after the stress test because blood cytokine profile variations have been reported under stress conditions.^43–45^ At baseline, we observed reduced plasma TNF- ɑ in the S7 recipient group compared to GF control (*p* =.09, One-way ANOVA, uncorrected Fisher’s LSD). Furthermore, transferring human microbiota from a frail donor to GF mice could also reduce the TNF-ɑ concentration in plasma (*p* =.06, One-way ANOVA, uncorrected Fisher’s LSD). Notably, TNF-ɑ concentrations in healthy donor recipient and Frail+S7 mice were significantly lower compared to germ-free control mice at baseline. Restraint stress caused a drop in plasma TNF-ɑ (*p* =.07, *t-test*) in germ-free mice, but this trend was not observed in all the other groups (Supplementary Figure S6a). There was no difference in MCP-1 concentration at baseline and after restraint stress between microbiome treatment groups (Supplementary Figure S6b). IL-6 levels were not significantly different between the groups at baseline, but restraint stress-induced IL-6 was observed in all groups, and stress-induced IL-6 was highest in frail donor recipient mice and was lowered by S7 treatment (Supplementary Figure S6c).

### Colonization by donor microbiota and the S7 species did not change the overall gut permeability, but reduced the colon epithelial permeability

We next investigated whether S7 administration modulated intestinal permeability in the humanized murine model *in vivo* at baseline and following acute stress by measuring plasma levels of orally administered FITC-dextran. There was no significant difference in gut permeability between groups at baseline (Kruskal-Wallis, *p* =.7958) or after 1 hour of restraint stress (Kruskal-Wallis, *p* =.6349) (Supplementary Figure S7a). The association of GF mice with either healthy or frail donor microbiota did not alter gut permeability compared with the GF control. Furthermore, S7 administration did not significantly alter the gut permeability of S7 recipient or Frail+S7 recipient mice compared with GF and frail donor recipient controls, despite a non-significant trend for all S7 recipient mice to show higher levels of basal (pre-stress) permeability. Notably, restraint stress significantly increased the gut permeability of humanized or S7-inoculated mice but not that of GF mice.

To characterize specifically colon epithelial permeability, we measured the efficacy of FITC diffusion across the epithelium in colon samples *ex vivo* (FITC flux). We observed reduced colon permeability in S7 recipient mice compared to their GF controls, which was also observed in other treatment groups (Supplementary Figure S7b). However, the S7 supplement in frail donor recipient mice did not affect colon permeability compared to frail donor recipient mice.

### Altered behavior in S7-treated frail donor recipients

**Figure 5.**
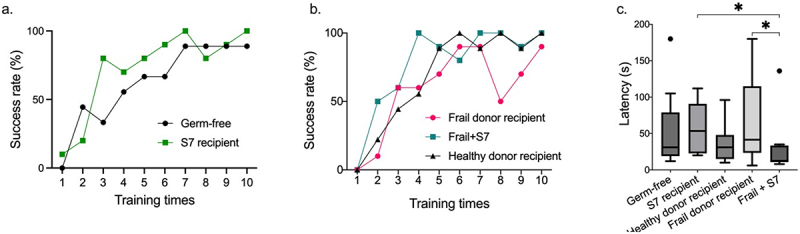
Supplementation with the S7 consortium improves behavior in frail donor recipient mice.

To test if S7 supplementation affected the gut-brain axis in frail donor recipient mice, we performed a series of behavior tests, including (1) grip strength test, (2) tail suspension test for depression-like behavior, (3) novel objective recognition test for cognitive function, (4) balance beam test for sensorineural balance and coordination, (5) Barnes maze test for spatial reference memory and learning. There was no significant difference between the treatment groups for grip strength, depression-like behavior, cognitive function, sensorineural balance, or coordination (Supplementary Figure S8a-d). However, S7 treatment of frail donor recipient mice improved the performance of female mice for grip strength and in the Barnes maze, which was not observed in male mice (Supplementary Figure S9). We observed improvement over time in all groups in the Barnes maze test, reflected in the increased number of individuals who were able to finish the test within 3 min (success rate) from training day 1 to the test day (Figure 5a, b). Notably, GF and frail donor recipients treated with S7 showed higher success rates than their GF and frail donor recipient controls (Figure 5a, b). On the test day, frail donor recipient mice with S7 treatment demonstrated improved spatial memory and learning ability (*p* =.03, one-way ANOVA post hoc Holm-Sidak) compared to frail donor recipient mice without S7 treatment (Figure 5c). These data suggest that S7 supplementation, which predominantly increased *B. intestinihominis* abundance, can potentially improve spatial memory and learning ability in frail donor-recipient mice.

Spatial reference memory and learning ability were assessed via Barnes Maze. Mice were trained for 3 d and each training day included three training trials, lasting 3 min to allocate the submerged platform spatially. A latency of 3 min was used as a cutoff for the success rate. a. Success rates of S7 recipient mice and their germ-free controls at each training time b. The success rate of frail donor recipient mice treated with S7 (Frail+S7) compared to their frail/healthy donor recipient controls c. Frail donor recipient mice treated with S7 species (Frail+S7) were significantly faster to reach the target hole than frail donor recipient controls on the test day (*p* = .03). Data were analyzed using a one-way ANOVA post hoc Holm-Sidak. Box plots include the median line and the box denotes the interquartile range. Whiskers indicate the 1.5 interquartile range. Dots represent the outliers. *P < .05, **P < .01. *n* = 9 (Germ-free), *n* = 10 (S7 recipient), *n* = 9 (Healthy donor recipient), *n* = 9 (Frail donor recipient), *n* = 10 (Frail + S7).

## Discussion

An accumulation of evidence indicates that the gut microbiota is an environmental modulator of aging-related health loss, and microbiota modulation or restoration has been proposed as an effective strategy for promoting healthy aging. However, precision microbiome manipulation in humans is technically challenging, expensive, and requires regulatory hurdles to be overcome, collectively making it highly desirable to be able to pre-screen for promising strains. It is, therefore, imperative to establish a valid animal model to generate mechanistic understanding of host–microbe interactions in the aging process. In this study, we used a human microbiota-associated (HMA) model to evaluate whether engraftment of a healthy or frail human microbiota could recapitulate host frailty-related phenotypes, and this model was used to investigate the effect of a seven-species consortium (the “S7”) as a microbiome-based therapeutic strategy for healthy aging. Metabolomic analysis revealed higher acetate levels in the feces of healthy donor recipient mice compared with that of frail donor recipient mice (Supplementary Figure S4). Consistent with the metabolic data, acetate-producing bacteria,^46^ including *Prevotella* spp. and *Bifidobacterium* spp., were present in the healthy donor recipient mice while absent in the frail donor recipient mice (Supplementary Figure S2).

While a previous study reported that fecal microbiota transplantation of frozen samples into GF mice resulted in the successful transfer of 85% of genera after 1 week,^47^ only 51% and 66% of frail and healthy donor genus-level taxa were transferred into mouse recipients 1 week after transplantation in our current study. This difference in transplantation efficacy could be due to the lower microbial diversity of the elderly healthy – and frail – donor we recruited. Lower engraftment efficacy of frail donor microbiota could be due to lower alpha-diversity and a low *Prevotella* to *Bacteroides* ratio compared to healthy donor microbiota.^48^ Furthermore, the germ-free mouse gut is characterized by increased mucin production, higher pH value, urea and oxygen levels, and low or absent short-chain fatty acids, substantially affecting gut microbiota colonization.^49–51^ Even though previous studies have shown a single dose of FMT is enough to replicate human microbiota in germ-free mice,^12,47^ some studies have highlighted that multiple gavage instead of a single dose increases the number of detected taxa,^52,53^ which should be considered in future studies to improve the engraftment rate of the elderly microbiota. It may alternatively be necessary to trial other preclinical models for human microbiome–frailty interaction if engraftment efficiency or readouts in a murine germ-free model prove limiting.

Using the selected readouts for several physiological systems, we also found that the human frailty phenotype could not be transmitted to GF mice by microbiota transfer, even though the engraftment of healthy or frail donor microbiota resulted in distinct microbiota in recipient mice. It has been reported that taxa that colonize in recipient animals may not engage in the host–microbe interactions that drive “native” disease-associated alterations.^34,35,54^ The engraftment in HMA mice with healthy or frail-associated microbiota may simply reflect the adaptation of very different microbial communities to a new gut environment rather than the human donors’ pathological states, thereby masking disease signatures. The transplantation of “healthy” and “frail” donor microbiota resulted in distinct microbiota in recipient mice at week 5 despite no difference in alpha diversity (Figure 2). To replicate the frailty-related phenotype in recipient mice, the engraftment of frailty-related taxa holds greater importance than alpha diversity which is useful in describing the microbiome states.^23^ The successful engraftment of two frailty-positive-related taxa, *Coprobacillu*s and *Parabacteroides*, in frail donor recipients but not in healthy donor recipients may play a role in replicating frailty-related phenotype (Supplementary Information). However, the inefficient engraftment of two frailty-positive-related taxa, *Escherichia-Shigella* and *Streptococcus*, could be one of the reasons why there were no measurable differences in frailty-related phenotypes (Supplementary Table S11), including inflammation, grip strength, and cognitive function, between frail and healthy donor microbiota recipients. Furthermore, the lack of changes in gut permeability in this study could hinder the crosstalk within the microbiota-gut-brain axis.

With a view to modeling the modulation of the gut microbiome of older people, we identified seven healthy aging-related species and supplemented GF and frail donor recipient mice with those frailty-negative-associated S7 species (Figure 2a). Consistent with previous probiotic studies^55–57^, S7 supplementation as a live biotherapeutic consortium in frail donor recipient mice did not change the alpha-diversity. Five S7 species were detected in GF mice at week 5 using 16S rRNA-specific primers. However, S7 supplementation in frail donor recipient mice increased only the abundance of *Barnesiella intestinihominis*, suggesting that S7 species encounter a higher degree of colonization resistance in the humanized mice than in the GF mice. We also observed the loss of donor-originated S7 species in the murine recipients (Figure 4a). *Alistipes putredinis* was detected in the healthy donor but failed to colonize in the murine gut at weeks 1 and 5. In another case, *Dorea longicatena* from a healthy donor was detected in the mouse gut microbiota at week 1 but was absent at week 5. Similarly, frail donor-originated *Eubacterium rectale*, *Faecalibacterium prausnitzii*, and *Roseburia hominis* failed to colonize in the mouse gut. The distinct engraftment efficacies of the same species in different microbial communities suggest that the interaction of whole microbiomes and administered strains is crucial for successful engraftment. The loss of donor-originated S7 species also indicates the challenges of recovering administered strains.

Changes in the gut microbiota might influence the bidirectional signaling between the gastrointestinal tract and the brain, thus modulating behavior.^58,59^ *B. intestinihominis* has been reported to be more abundant in high-functioning older adults (aged above 70) compared with low-functioning adults, and the transfer of high-functioning donor microbiota into GF mice improved their grip strength^60^. Our data reveal improved spatial memory and learning ability in frail donor recipient mice receiving S7 treatment, mainly induced by increased *B. intestinihominis*, which may provide a tractable reductionist model for future studies seeking to identify microbial effectors and host mechanisms.

Supplementation of S7 in GF mice significantly increased fecal acetate levels (Supplementary Figure S5), confirming *in vivo* the reported ability of *B. intestinihominis*^61^ and *D. longicatena*^62^ to produce acetate. Furthermore, S7 supplementation reduced the plasma TNF-ɑ concentration compared to the GF controls, which is consistent with reduced colon permeability in S7 recipient mice, suggesting the beneficial effects of the S7 consortium. Although it was previously reported that the transfer of gut microbiome from old mice into young mice affects inflammaging, the reduction in TNF-ɑ in S7-supplemented animals we observed may have been related to the stress test response. The S7 consortium did not change the short-chain fatty acids (SCFA) level of frail donor recipients. The lack of observed effect of the S7 consortium on SCFA in frail donor recipients could be attributed to one or more of the following: i) SCFA is the product of fermentation of gut microbiota, which is regulated by microbe–microbe and microbe–host interactions, and frail microbiota is a complex community, ii) SCFA can be directly used as energy and carbon sources by the murine host,^63^ and iii) difficulties of SCFA measurement in the feces due to their volatility and hydrophilicity. The S7 supplementation in frail donor recipient mice did not change the overall gut permeability and colon permeability compared to frail donor recipients. However, the stress-reduced IL-6 was higher (*p* = .08) in frail donor recipients compared to GF mice, which was not observed in frail donor recipients with S7 supplementation (Frail + S7). The inconsistent change of stress-reduced IL-6 between Frail + S7 and frail donor recipients could be explained by the permeability changes in other intestinal locations. It has also been reported that humanized GF mice display reduced immune stimulation compared to mouse microbiota-transplanted mice.^64^ Moreover, GF mice are known to have an underdeveloped immune system due to the lack of gut microbiota,^65,66^ further highlighting the challenge of adapting this model for mechanistic studies.

Because of the usage of single donors in the preclinical model, we could not test the effect of different donors/microbiome composition within healthy and frail groups and compare them at higher taxonomic resolution (i.e., strain level) for colonization efficiency and phenotype transmission. Although some studies seeking to transfer human phenotypes to experimental animals via the microbiome have used pooled samples from multiple donors, we chose not to do this because it produces artificial trophic networks and functional redundancies. Although we carefully selected donors that were representative of clinical phenotypes and the corresponding microbiome type, using single donors for the two groups is a limitation we acknowledge. We also recognize that the current study does not prove the causality of improved spatial reference memory and learning ability. Further studies will be needed to understand the role of *B. intestinihominis* in improving these readouts, supported by a wider battery of tests targeting this aspect of cognitive function. Overall, this study demonstrates the difficulty of replicating the frailty phenotype in the humanized GF mouse model and provides essential information for design considerations in elderly microbiota transplantation studies.

## Methods

### Identification of healthy aging-related taxa for constructing a synthetic consortium

Diet is a significant modulator of the gut microbiota at all life stages and one that particularly drives alteration of the gut microbiota in aging. Our previous study investigated the effect of a 1-y Mediterranean diet intervention (NU-AGE diet) in a large cohort of more than 1200 elderly individuals aged 65–79 y, distributed across five countries (UK, France, Netherlands, Italy, and Poland). This study revealed that pre-frail elderly subjects receiving a 1-y NU-AGE diet experienced delayed onset of further frailty by modulating the gut microbiota.^24^ Taxa enriched by adherence to the NU-AGE diet were referred to as “diet-positive” taxa. The NU-AGE study thus provided a short list of “diet-positive” taxa that have the potential to promote healthy aging, including *Faecalibacterium prausnitzii*, *Eubacterium rectale*, *Roseburia hominis*, *Prevotella copri*, *Blautia* spp., and *Clostridium* spp.

Aging is associated with multiple diseases, and a previous study in our lab also identified aging-associated microbiome disease markers across five major diseases in a multi-cohort dataset, including more than 2,500 individuals from different countries of North America, Europe, and Asia.^11^ In Ghosh *et al*., we reported reduced abundance of *Alistipes putredinis*, *Barnesiella intestinihominis*, *Coprococcus catus*, *Dorea longicatena*, and *Eubacterium rectale* in at least two diseases.^11^ Furthermore, *F. prausnitzii* has been negatively associated with frailty in a twin study.^26^ Combining the overlapping results of disease- and diet-associated taxa, a candidate group of species (that we refer to here as the S7), namely *Alistipes putredinis*, *Barnesiella intestinihominis*, *Coprococcus catus*, *Dorea longicatena*, *Eubacterium rectale*, *Faecalibacterium prausnitzii*, and *Roseburia hominis*, was selected for further analysis.

### Relative abundance of the S7 taxa in the ELDERMET cohort and IMNGS database

The ELDERMET shotgun data were retrieved from the European Nucleotide Archive (ENA) with the project accession number PRJEB37017. Samples with available Functional Independence Measure (FIM)^67^ and Barthel Scores^68^ were selected for the S7 profile analysis (Supplementary Table S6). Selected samples comprise 188 elderly Irish individuals (aged between 64 and 102 y of age), with 135 subjects living in the community and 55 subjects living in Residential care (Long-stay). FIM values equal to or less than 90 are classified as frail,^67^ values between 90 and 126 are classified as mid-frail, and values equal to 126 are defined as non-frail/healthy.

The occurrence and prevalence of 16S rRNA gene sequences related to the S7 taxon were analyzed with the Integrated Microbial Next-Generation Sequencing (IMNGS) platform.^37^ All Sequence Read Archive (SRA) amplicon sequence datasets with the Origin from the human and mouse gut were selected for further analyses. A 99% sequence similarity cutoff was used to identify S7-related 16S rRNA gene sequences in the selected dataset, which includes 41,920 human and 19,703 mouse samples.

### Donor fecal sample and inoculum preparation

The “healthy” and “frail” donors (n = 1) were selected from a subset of the well-phenotyped ELDERMET subjects^69^ who live in community or long-stay residential care. Claesson et al. reported that gut microbiota of the ELDERMET cohort formed groups, correlating with residence location and diets. Furthermore, the clustering of subjects by diet separated them by the same residence locations and microbiota groupings.^3^ The average age of ELDERMET is 78 ± 8 y, with a range of 64 to 102. Regardless of age variance, the gut microbiota of ELDERMET cohort exhibited distinct clustering patterns correlated to frailty.^3^ The following exclusion criteria were used for donor selection: usage of antibiotics there months before the sampling; usage of immunosuppressive or anti-inflammatory drugs or any drug affecting intestinal mobility; participants on warfarin; participation in another clinic or intervention study involving investigational drugs, including investigational prebiotic, prebiotic or symbiotic preparations in the preceding 6 months.

The “healthy” and “frail” donor samples were selected based on consideration of their clinical measurements in ELDERMET project, as well as microbiome configuration. The diagnosis of frailty was provided by their consultant gerontologist based on combined evaluation of the body mass index, calf circumference, systolic blood pressure, Barthel index,^68^ functional independence measurement (FIM), mini-mental state exam, mini-nutritional assessment, geriatric depression test, and Charlson index of comorbidity.^3^ Barthel Index, Charlson co-morbidity index, and handgrip strength at sampling were also used as confirmatory references for donor selection (Suplementary Table S1). The microbiome composition of candidate donors was determined by 16S rRNA gene sequencing. We also compared the microbiota of candidate donors (from clinical evaluation) to the microbiomes previously analyzed ELDERMET samples to ensure the typical community or long-stay microbiota profiles were present in the donors (Supplementary Figure S1). Eventually, one “healthy” and one “frail” donors were selected for fecal microbiota transplantation (FMT). Upon collection, fecal samples were transferred to an anaerobic cabinet immediately after collection and homogenized in reduced sterile phosphate-buffered saline (PBS) with 20% glycerol, then stored at −80°C for transplantation. Additionally, the survival rate of donor taxa was checked by plating before and after the storage to ensure no taxa loss caused by the preservation method.

The S7 culture is constituted of a total of seven bacterial species from the previously isolated and characterized the Microbiome Culture Collection 100 (MCC100)^27^ and S7 species were cultivated in a modified YCFA medium as described previously^27^ at 37°C in an anaerobic chamber under an anoxic atmosphere (90% N_2_, 5% CO_2_, 5% H_2_). S7 pure cultures were harvested in the late exponential growth phase and centrifuged to reach the maximum cell count. The cell counts of S7 species were measured by plating on YCFA agar. The final S7 culture mix consisted of *A. putredinis* (1.83 × 10^8^ CFU/ml), *B. intestinihominis* (5.0 × 10^8^ CFU/ml), *C. catus* (2.14 × 10^8^ CFU/ml), *D. longicatena* (3.28 × 10^8^ CFU/ml), *E. rectale* (1.15× 10^8^ CFU/ml), *F. prausnitzii* (4.0 × 10^5^ CFU/ml), *R. hominis* (4.5 × 10^7^ CFU/ml). Bacterial cell mixtures were kept at −80°C in 20% (v/v) glycerol/PBS until further use.

### Pre-clinical animal model

All animal protocols were approved by the Animal Experimentation Ethics Committee at University College Cork and by the Health Products Regulatory Authority (HPRA) of Ireland in accordance with EU Directive 2010/63/EU (HPRA Project authorization number AE19130/P147).

Six-month-old C57BL/6 mice from the gnotobiotic line bred at the APC Microbiome Institute’s Germ-Free Platform were used for the experiment as fecal microbiota transplantation (FMT) from aged mice to young mice has been proven to be sufficient to change microbial metabolites in the host and to cause a reduction in cognitive function.^39,70^ We ensured gender balance within the experimental groups by incorporating an equal number of male and female mice (Supplementary Table S9). All the mice were group housed 3–4 per cage in the same temperature and humidity-controlled animal room with a 12 h light: dark cycle and were maintained on *ad libitum* sterile standard chow and water unless otherwise noted. Mice were colonized by pipette dosing with 200 µl of FMT (10^9^ CFU/ml) from either a single healthy or frail elderly donor (or sterile PBS as vehicle control). The “healthy” donor sample was used to humanize all the mice in the group “Healthy donor recipient,” and the “frail” donor sample was used to humanize all mice in groups ‘Frail donor recipient” and “Frail + S7”. Following 1 week of colonization, mice were gavaged once a week (for 4 weeks) with 200 µl of either the S7 treatment or vehicle control. After transplantation, mice that received the same treatment were co-housed in individually ventilated cages for the duration of the experiment. The cages, food, bedding, and water were sterilized by autoclave to maintain germ-free condition. Fecal samples were collected 1 week after FMT inoculation and 4 weeks after S7 supplementation to assess the effects of FMT on recipient fecal microbiota and the influence of S7 treatment. Following 4 weeks of S7 supplementation, blood was collected for gut permeability and inflammation assessment (see sections on FITC measurement and cytokine assessment). Mice then underwent behavioral tests to assess physical and cognitive frailty, including grip strength, tail suspension, balance beam, and Barnes maze. Finally, mice were sacrificed to collect blood and tissues for further analysis.

### DNA extraction for 16S rRNA amplicon microbiota analysis

Fecal DNA was extracted using the DNeasy Blood & Tissue Kit (QIAGEN) according to the manufacturer’s instructions. The V3-V4 region of the 16S rRNA gene was amplified, and the amplicons were quantified with a Qubit dsDNA HS Assay Kit (Thermo Fisher Scientific). Sequencing was performed on an Illumina MiSeq Platform (2 × 300 bp reads) by the Eurofins Genomics Next-Generation Sequencing service (London, UK).

### High-throughput 16S rRNA gene amplicon analysis

Raw reads were used for quality filtering and trimming using DADA2 (version 1.24.0)^71^ with parameters trimLeft = 12, truncLen = 250, and maxEE = 2 in R (version 4.2.1). Only forward reads were further processed and used for downstream analysis due to decreased quality of the reverse reads, which can negatively affect sample inference in the DADA2 pipeline. Taxonomy assignment was conducted using the SILVA SSURef database release v. 138.^72^ Species were assigned using spingo v1.3.^73^ Raw read counts were used as input data for differential analysis using DESeq2.^38^

### Quantitative PCR (qPCR) of S7 16S rRNA genes and total 16S rRNA gene copies in donor feces

To investigate the engraftment of S7 in germ-free mice, the fecal DNAs from S7 recipient mice were subjected to quantitative PCR using primers specifically targeting the individual S7 16S rRNA gene (Supplementary Table S10). 16S rRNA-specific primers were designed using Primer3Plus.^74^ Standard curves were generated from genomic DNA extracted from S7 pure cultures. In order to quantify the total bacteria, DNA was extracted from human donor feces using the QIAamp DNA Stool Mini kit following the manufacturer’s instructions. The 515F/806 R primers were chosen to quantify total bacteria, and standard curves were constructed using the PCR product of the 16S rRNA gene of *E. coli*. The PCR product was purified using the QIAquick PCR Purification Kit (QIAGEN, Germany) and quantified with a Qubit dsDNA HS Assay kit (Thermo Fisher Scientific, Ireland). One PCR reaction (total volume of 15 μl) contains 0.75 μl of primers (10 μM), 7.5 μl SYBR Green I, 5 μl nuclease-free water, and 100 ng DNA template. PCR conditions were 95°C for 10 min, followed by 40 cycles of 95°C for 10 s, 55°C for 15 s, and 72°C for 15 s. Fluorescence signals were obtained at the end of each cycle. Amplification and detection were performed using the LightCycler 480 system (Roche, US). All qPCR assays were performed in duplicate, and the amplification efficiencies of all qPCR assays are >98%.

### SCFA and organic acid analysis

Mouse fecal pellets were diluted 1:5 (w/v) with water, vortex-mixed for 5 min and sonicated for 5 min. Homogenized samples were centrifuged for 10 min at 13,000 *g*. The supernatant was collected and filtered through a membrane filter (0.45 μm), and a volume of 20 μl sample was used for further measurement. Short-chain fatty acids (SCFAs) were determined with an Agilent 1200 HPLC system (Agilent Technologies, CA, USA) with a REZEX 8 µL 8% H + organic acid column (300 × 7.8 mm) (Phenomenex, CA, USA) operating at 65°C using 0.01 N H_2_SO_4_ as a solvent at a flow rate of 0.6 mL/min. Organic compounds were detected with a refractive index detector. Reference standards of SCFAs and organic acids, including glucose (10 mM), lactose (10 mM), lactic acid (10 mM), formic acid (10 mM), succinic acid (10 mM), propionic acid (10 mM), acetic acid (10 mM), butyric acid (10 mM), were used for data analysis.

### Cytokine measurement

Inflammation-related cytokines IL-6, CCL2 (MCP-1), and TNF-ɑ were assessed using the LEGENDplex Mouse Inflammation Panel (Mix and Match Subpanel) assay (BioLegend) according to the manufacturer’s instructions. Four tail blood samples from each group before (baseline) and after restraint stress were collected using a heparinized capillary tube for cytokine measurement. Samples were run in duplicate. Data analyses were performed using BioLegend’s LEGENDplex™ data analysis software.

### Intestinal permeability assay by FITC-dextran

#### *In vivo* FITC permeability

Intestinal barrier function was assessed using fluorescein isothiocyanate-labeled dextran (FITC-D) (FD4, Sigma Aldrich, Ireland) as previously described.^75^ Briefly, the mice were fasted overnight and orally gavaged with FITC-D (600 mg/kg, 80 mg/ml) the next morning. One hour following gavage, mice were restrained, and tail blood samples were collected using a heparinized capillary tube for baseline permeability. Blood samples for stress-induced permeability were collected following 1 hour of restraint stress. Blood samples were kept on ice and centrifuged to collect plasma. Plasma samples were stored at −20°C for FITC measurement. FITC in plasma was measured in *Nunc™* 384-well Optical Bottom plate (Thermo Fisher Scientific) at 485 nm excitation and 535 nm emission wavelengths using a multi-mode plate reader (Victor 3, Perkin Elmer). Serial dilutions of FITC in PBS were used for the standard curve.

#### *Ex-vivo* FITC colonic permeability

Fresh colon segments (1–2 cm) were collected at the end of the experiment to examine the colonic permeability. Colon tissues were washed with PBS and were mounted into vertical NaviCyte diffusion chambers with 4 mm round aperture (0.126 cm2 exposed tissue area). No seromuscular stripping was performed. Then, 4.0 mL of Krebs buffer (1.2 mM NaH_2_PO_4_, 116 mM NaCl, 4.8 mM KCl, 1.2 mM MgCl_2_, 25 mM NaHCO_3_, 2.5 mM CaCl_2_, and 10 mM D-glucose) was added to both the mucosal and serosal chambers. Chambers were kept at 37°C and continuously supplied with carbogen (95% O2 and 5% CO2). To assess the transepithelial permeability, 4 KDa FITC-dextran (FD4, Sigma-Aldrich) was added to the luminal chamber (final concentration of 2.5 mg/ml). Samples (200 μl) from the serosal chamber were collected at 0 h, 1 h, 1.5 h, and 2 h.^76^ FITC absorbance was measured in 96 well plates as mentioned above. FITC mucosal-to-serosal flux was presented in µg/h/cm^2^.

### Behavioral tests

All behavior tests were conducted as the last tests (Figure 1) and scored blinded. Both the grip strength and tail suspension tests were conducted under sterile conditions in a biosafety cabinet. Due to the size of the Barnes Maze, novel object recognition, and balance beam tests, these tests were conducted outside of a sterile environment.

#### Novel object recognition (NOR)

To assess short-term recognition memory, mice were subjected to the NOR task as previously described.^77^ Animals were habituated to the room 60 min before the test. On the pre-trial day, mice were habituated to the empty, open arena (40 × 45 × 45 cm, L × W × H) in two habituation phases (10 min each, with 3 h time in between). The following day, mice were exposed for 10 min to two identical objects placed in the arena’s corners (acquisition phase). Three hours later, one of the familiar objects was substituted with a novel object, and the animal was allowed to explore the objects for 10 min (retention phase). The test was conducted under dimmed lighting (15 lux). Animal behavior was video recorded, and the exploration time of the objects was recorded. The discrimination index was calculated according to the formula: (*t*(novel)-*t*(familiar))/(*t*(novel)+*t*(familiar)).

#### Balance beam

Mice were placed onto a wooden balance beam (12 mm wide, 125 cm long, elevated 60 cm above the floor), and the time to reach the escape box (20 × 20 × 20 cm) at the end of the beam was recorded. If animals did not readily walk across the beam, they were gently encouraged at 2, 3, 3:30, 4, and 4:30 min. The test was ended if the mice failed to cross the beam in 5 min. A net was placed below the beam in case of falls, and if an animal fell, it was placed back on the starting end of the beam, and the number of falls was recorded.

#### Grip strength

Forelimb grip strength was measured using a grip strength meter (Ugo Basile, Italy), which measures the peak pull force (g) exerted by the mice on the grasping apparatus. Mice were held with their forelimbs on the grip bar and were gently pulled backward along a horizontal plane while they grasped the bar. The forces from five consecutive trials were recorded and used for analysis.

#### Barnes Maze

The Barnes maze is used to assess spatial reference memory and learning and was conducted as previously described.^78^ Briefly, mice were trained over 3 d, with three trials per day, each lasting 3 min to allocate the submerged platform spatially. The latency to find the correct hole was analyzed by comparing the average of three trials across each training day to evaluate learning performance.

#### Tail suspension

The tail suspension test is used for screening potential antidepressant therapeutics.^79^ Mice were suspended by their tails from a horizontal bar for 6 min, which resulted in initial escape-oriented behaviors and immobile posture after several minutes. The time to the first immobility and the total immobility time were recorded for further analysis.

### Statistical analyses

Statistical analyses were conducted using R and GraphPad Prism v.9.4.1 (GraphPad Software). Outliers were excluded using the ROUT method with q = 1%.^80^ Figures with outliers were presented in Supplementary Figure S10. Normality analyses were conducted using the Shapiro–Wilk test. Unless otherwise indicated, nonparametric data were analyzed by Kruskal–Wallis post-hoc Dunn’s and parametric data by one-way ANOVA post-hoc Holm-Sidak. The similarity between the samples was calculated with Spearman’s correlation (ρ) using cor() in R (version 4.2.1).^81^ All tests were two-sided, and statistical significance was set at *P* <.05.

## Supplementary Material

Supplemental Material

## Data Availability

All 16S rRNA gene sequence data are deposited to the Sequence Read Archive (SRA) under BioProject accession PRJNA943425.
