# Supplementary material for: Engraftment of aging-related human gut microbiota and the effect of a seven-species consortium in a pre-clinical model
Source: Gut Microbes. 2023 Nov 27;15(2):2282796. doi: 10.1080/19490976.2023.2282796 (PMC10854441; doi:10.1080/19490976.2023.2282796)
Supplement: Supplemental Material [file KGMI_A_2282796_SM5953.zip › Supplemental materials/Supplementary_figures_.docx]

**Engraftment of aging-related human gut microbiota and the effect of a seven-species consortium in a pre-clinical model**

**Supplementary figures**


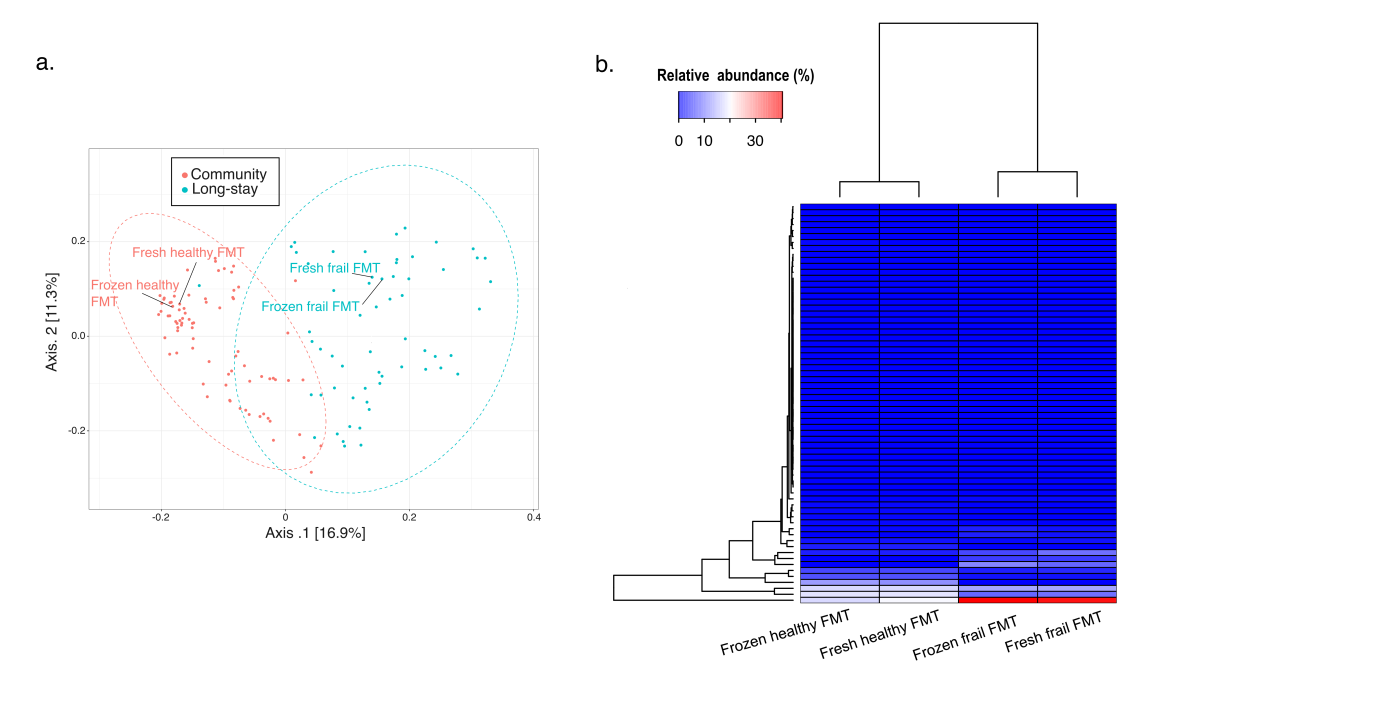


**Supplementary Figure S1**. **a.** Beta diversity comparisons based on Jensen-Shannon Divergence (JSD) values of the microbiota of community and long-stay elderly subjects. Samples are from a subset ELDERMET cohort with 79 subjects from community and 59 from long-stay residential care. Frozen and fresh samples from selected donors are labelled. **b.** Heatmap of taxa at family level. The heatmap shows that all taxa present in fresh fecal samples can be detected in frozen fecal samples at family level.


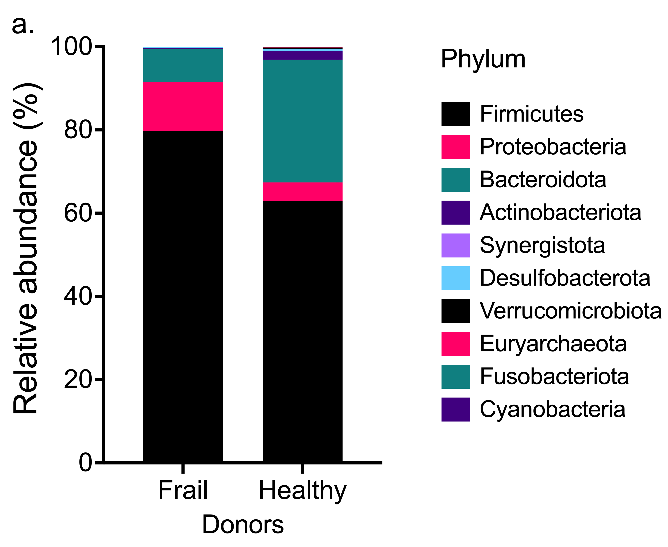


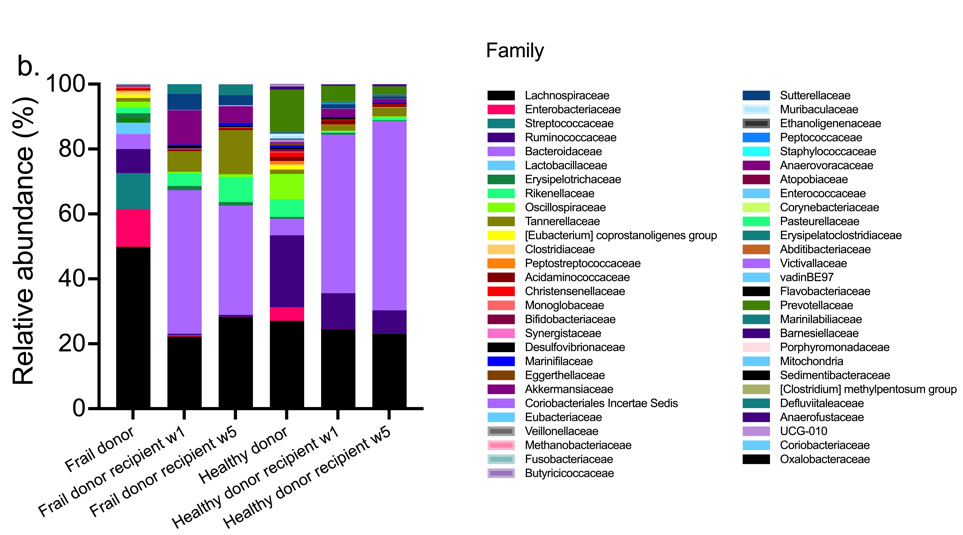


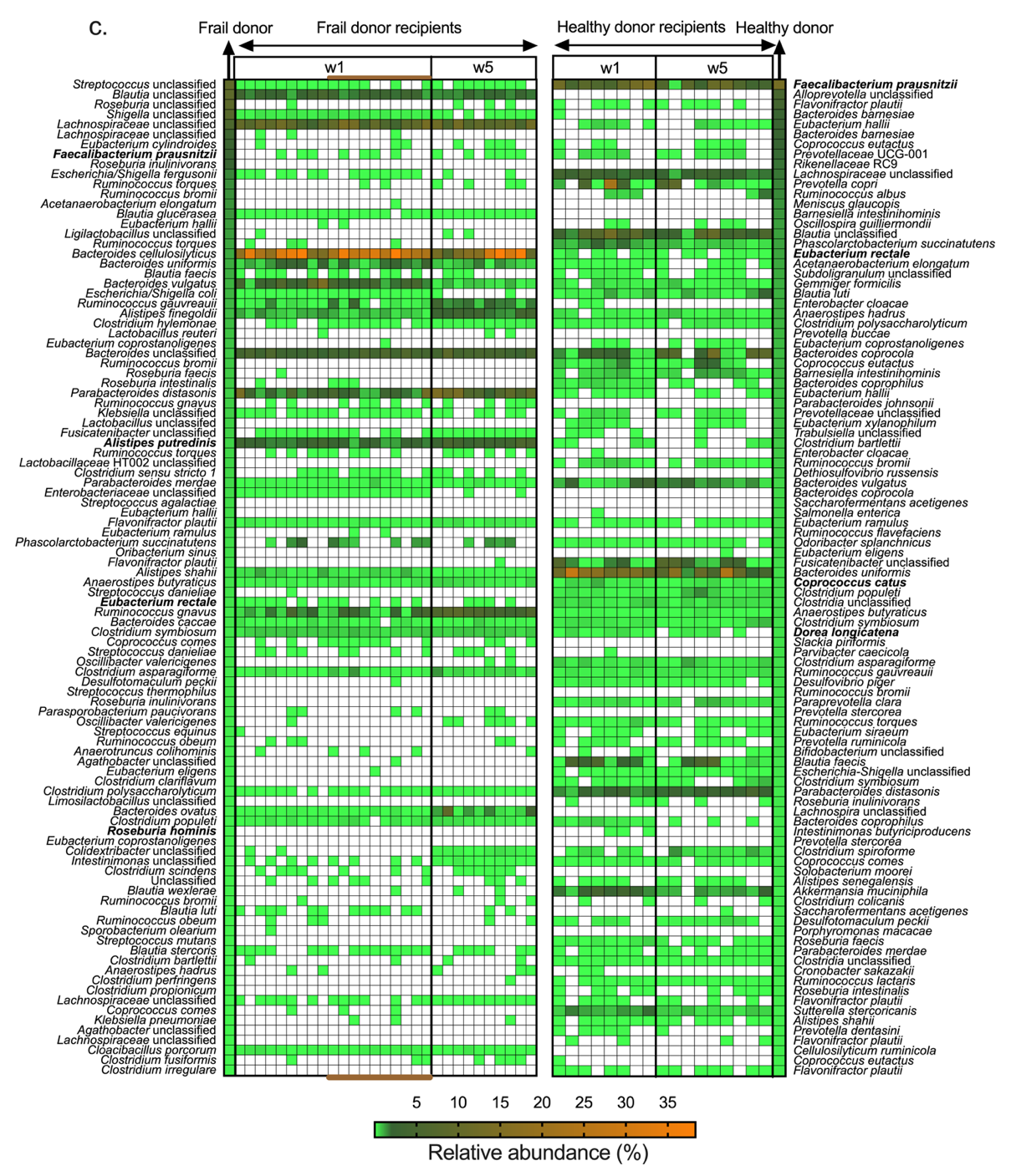


**Supplementary figure S2.** **a.** Comparison of frail and healthy donor gut microbiota composition at phylum level **b.** Relative abundance at the family level of FMT donors and recipients. Data are the mean relative abundance of all samples in each group except for each donor (n=1). **c.** Species engraftment of human gut microbiome taxa in the murine gut. The heatmap shows the presence and absence of the top 100 donor microbiota species in the murine recipients at weeks one and five following FMT. Fecal samples from frail or healthy donor microbiota recipient mice were collected at weeks one and five post-FMT for 16S rRNA gene amplicon sequencing. The most abundant 100 species in frail or healthy donors (n = 1) were identified and presented in descending order. The S7 species are marked in bold. Each column represents an individual (donor human or mouse recipient, as labeled above). n = 19 (Frail donor recipient w1), n = 10 (Frail donor recipient w5), n = 8 (Healthy donor recipient w1), n = 9 (Healthy donor recipient w5). Frail donor recipients at w1, further treated with S7 at week five, were marked with a brown line (n = 10).


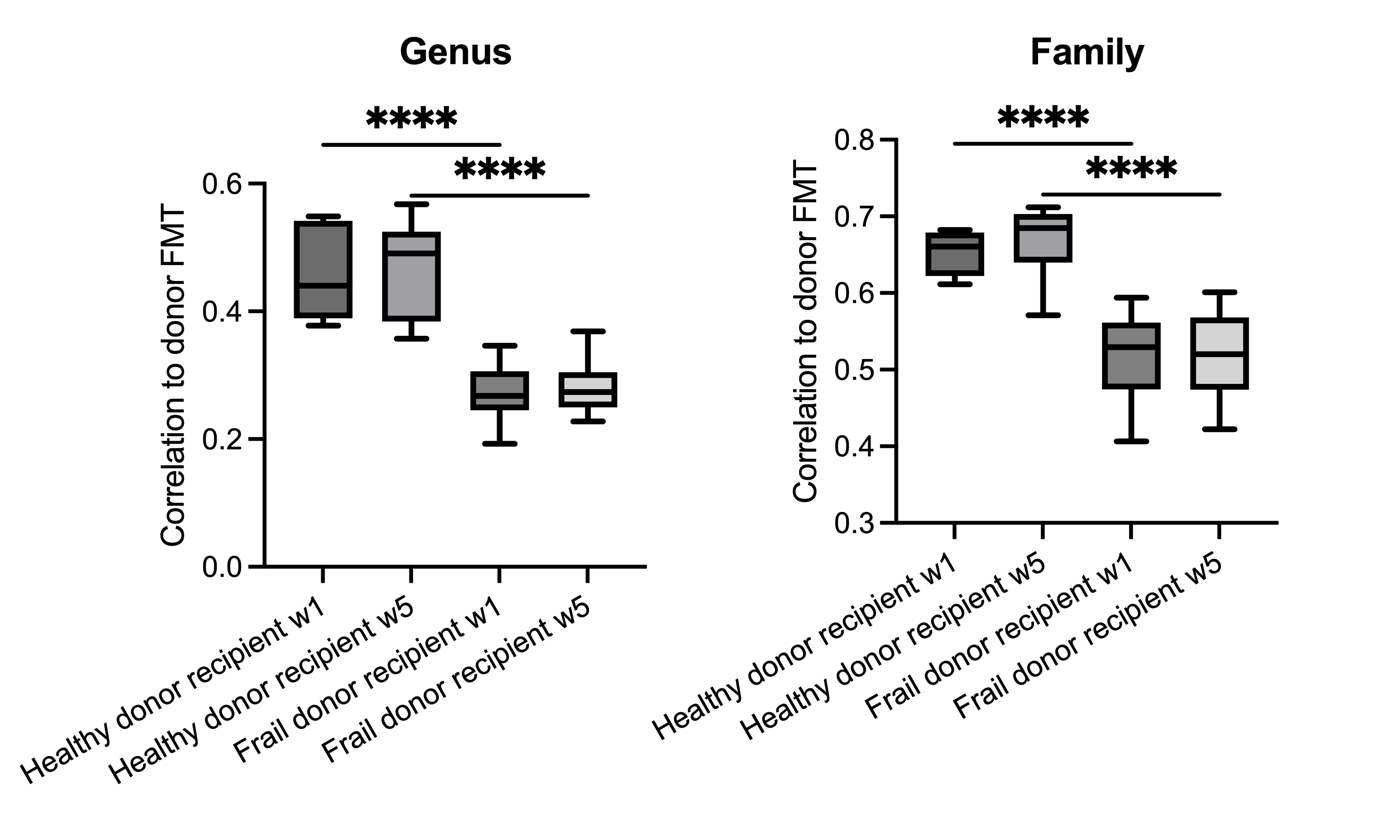


**Supplementary figure S3.** Correlation of recipient microbial compositions to that of corresponding donor at genus and family level at weeks one and five. Box plots with median lines showed the minimum and maximum values. n = 19 (Frail donor recipient w1), n = 10 (Frail donor recipient w5), n = 8 (Healthy donor recipient w1), n = 9 (Healthy donor recipient w5). One-way ANOVA post hoc Holm-Sidak. **** *P* < 0.0001.


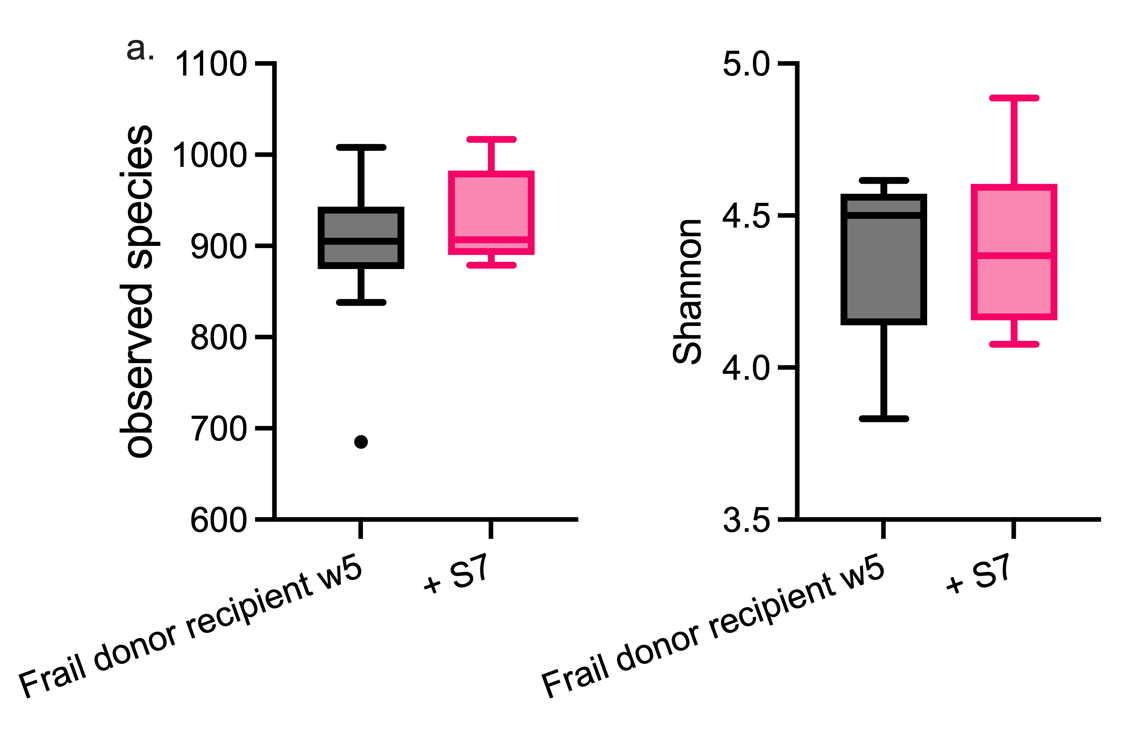


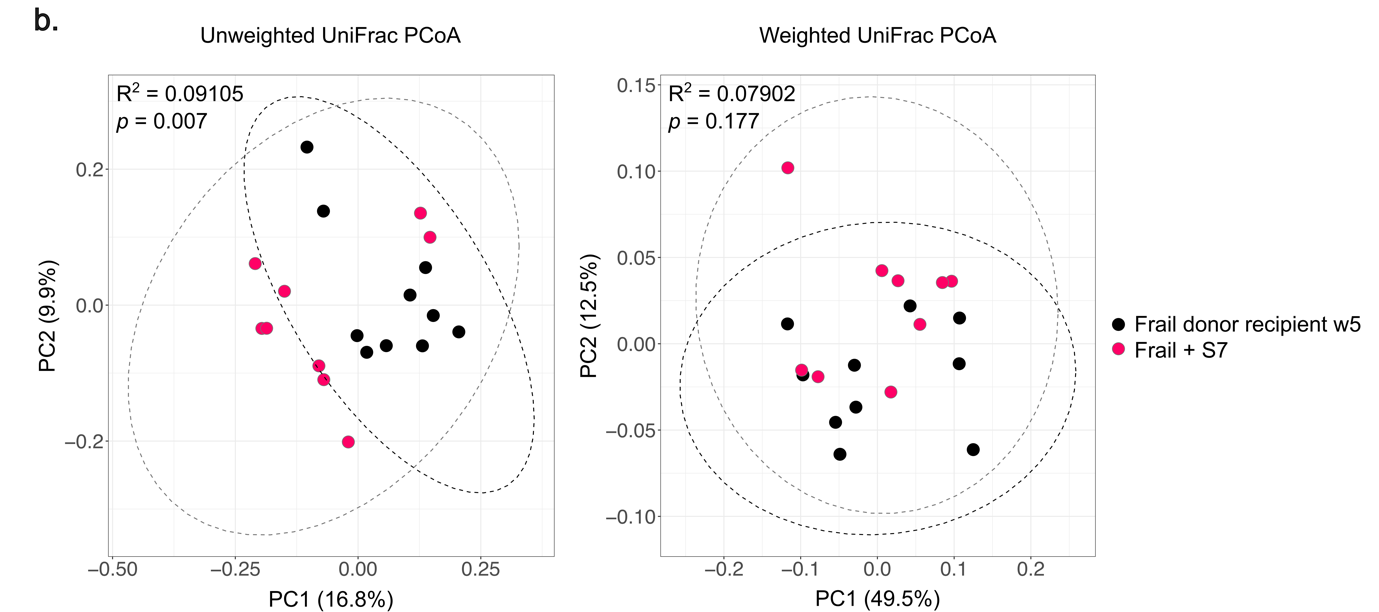


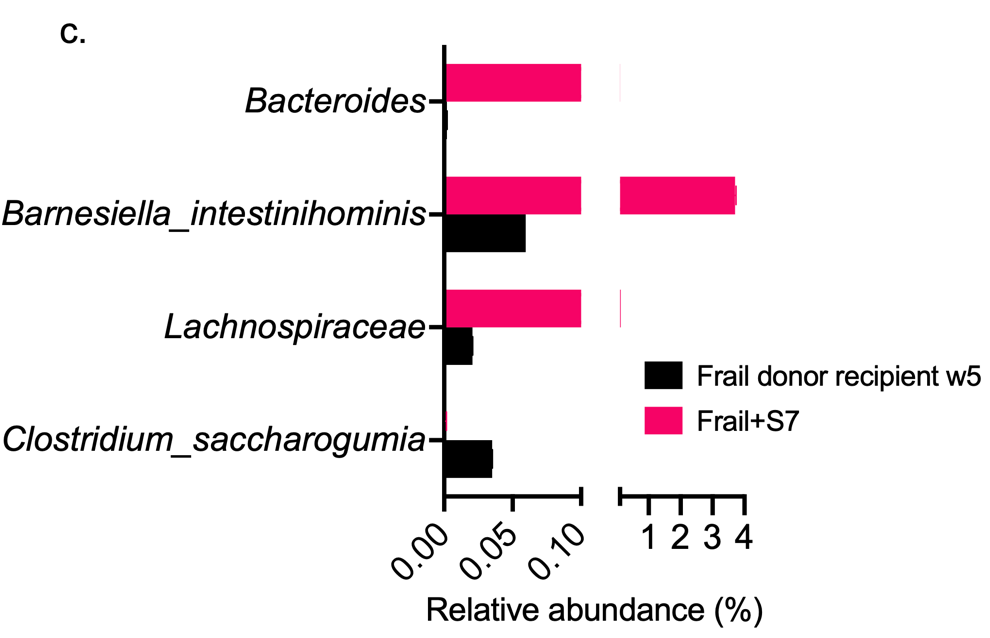


**Supplementary figure S4.** **a.** Boxplot of alpha-diversity indices. α-diversity was measured using Observed Species and Shannon Index. The horizontal line inside the boxes defines the median value, and outliers are shown as dots. **b.** PCoA derived from unweighted and weighted UniFrac distances of frail donor recipient and frail+S7 groups. **c.** Differential taxon abundance between the frail donor recipient and frail+S7 groups. Statistically significant differences were determined using DESeq2 (*p*-value < 0.05). Bars are plotted with Mean ± SEM.


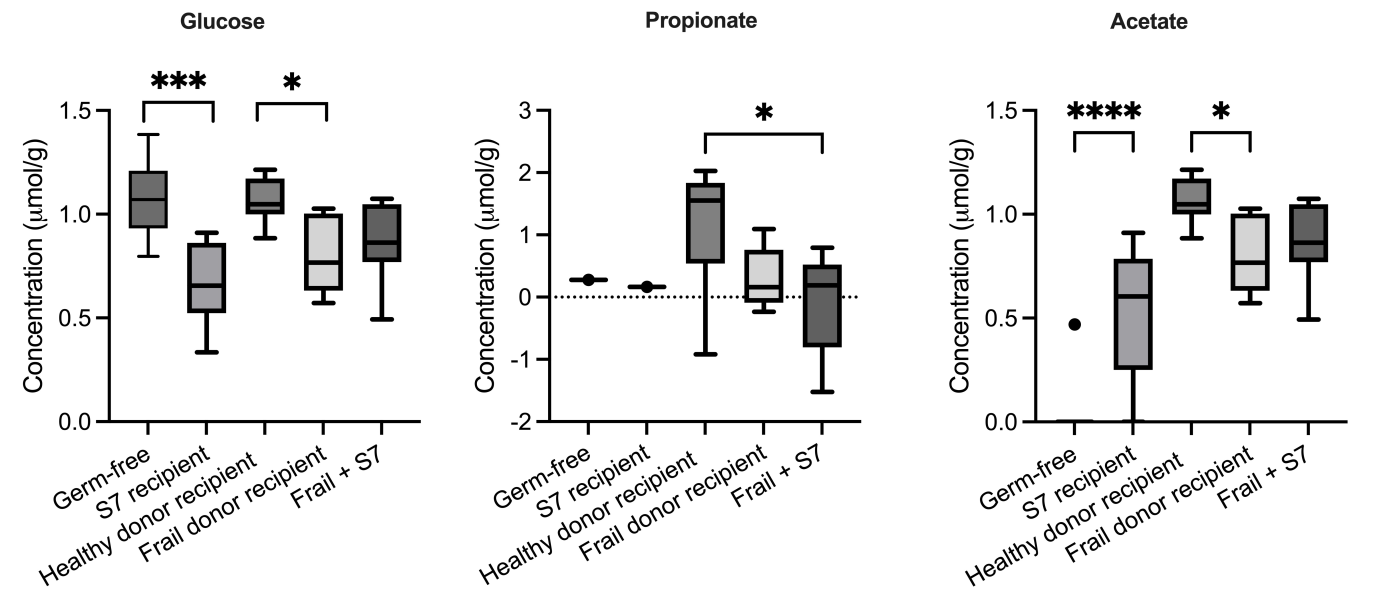


**Supplementary figure S5.** Measurement of short-chain fatty acid (SCFA) and organic acids levels in the murine recipient fecal samples at week 4. Fecal SCFA and organic acid concentrations were determined by HPLC and are expressed in μmol/g feces (log-transformed). S7 recipients were compared to Germ-free controls, and a pairwise comparison was performed between Healthy donor recipient, Frail donor recipient, and Frail donor recipient treated with S7 (Frail+S7). Only SCFA and organic acids with significant differences are shown. Box plots visualizing the distributions of log-transformed SCFA concentrations. one-way ANOVA post hoc Holm-Sidak, **P* < 0.05, ****P* < 0.001. n = 9 (Germ-free), n = 10 (S7 recipient), n = 8 (Healthy donor recipient), n = 10 (Frail donor recipient), n = 10 (Frail + S7).


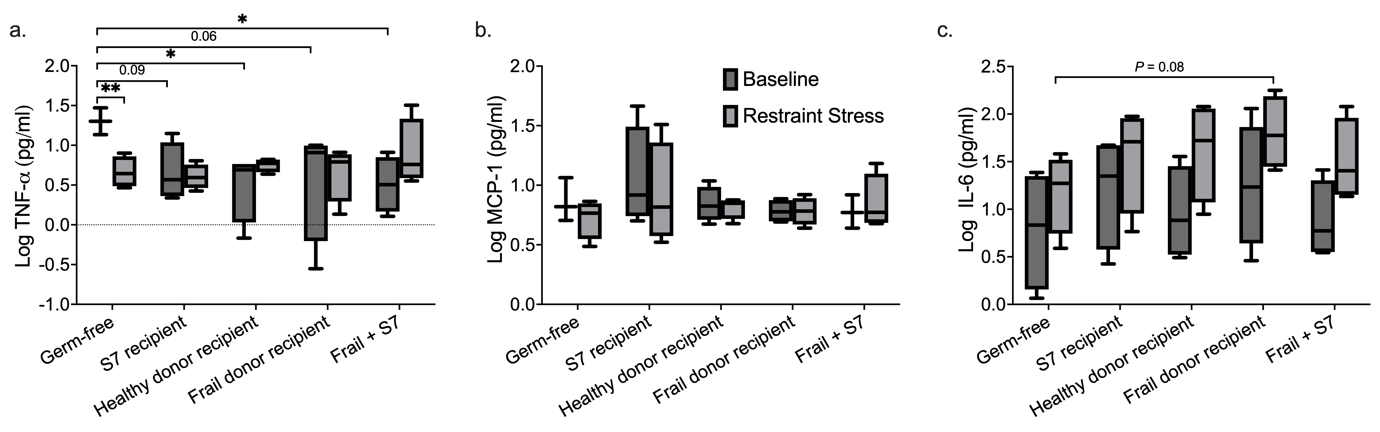


**Supplementary figure S6. Plasma pro-inflammatory cytokine levels of germ-free and recipient mice** **(a.)** Tumor necrosis factor-alpha (TNF-ɑ), **(b.)** monocyte chemoattractant protein-1 (MCP-1), and **(c.)** interleukin‐6 [(IL-6)](https://agsjournals.onlinelibrary.wiley.com/doi/abs/10.1111/j.1532-5415.2011.03570.x) concentrations in plasma were measured before (baseline) and after restraint stress (stress-reduced). Box plots visualizing the distributions of log-transformed cytokine concentrations. Statistical significance between baseline and restraint stress was determined using paired *t*-test (n = 4). Differences between treatment groups were identified by uncorrected Fisher’s LSD test in multiple comparisons after one-way ANOVA. ***P* < 0.01, **P* < 0.05.


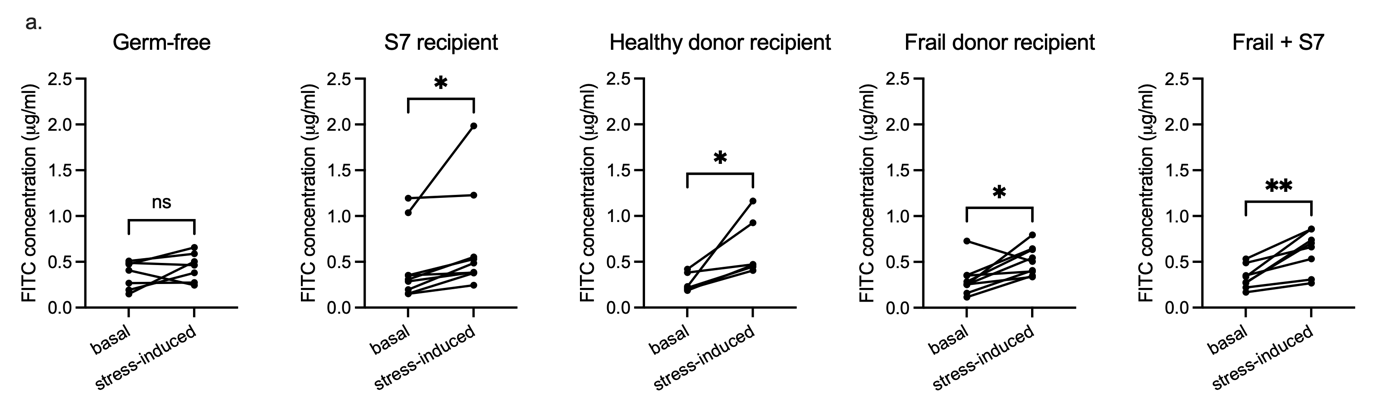


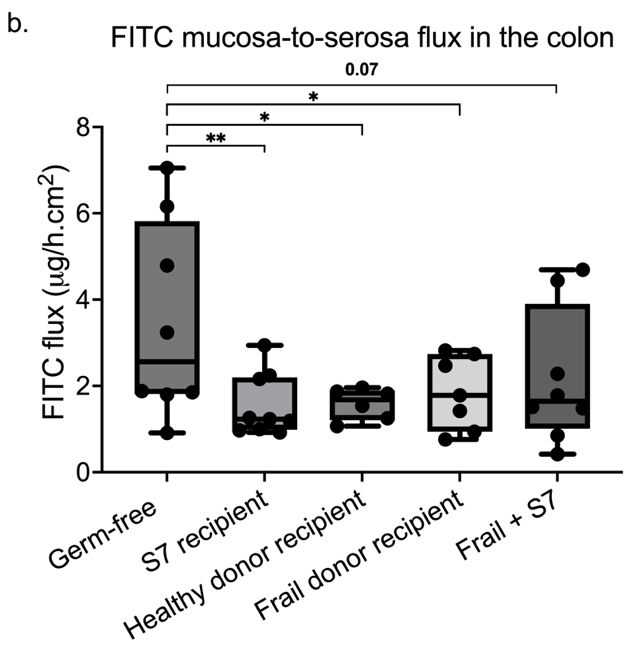


**Supplementary figure S7. Effect of microbiome treatment on epithelial permeability. a.** Epithelial permeability before (basal) and following 1h of restraint stress (stress-induced) was assessed by measuring the efficiency of macromolecular diffusion across the epithelium *in vivo* using the FITC-D test. The epithelial permeability did not differ between groups at either time point (*P* > 0.05). Restraint stress increased the gut permeability of S7-, healthy donor-, frail donor-, and Frail+S7 recipient mice (paired-wise *t*-test). Each dot represents one animal. n = 6 (germ-free), n = 6 (S7 recipient), n = 5 (Healthy donor recipient), n = (Frail donor recipient), n = (Frail+S7) **b.** The transepithelial flux of 4kDa FITC-dextran, detected in the serosal chamber after 120 minutes of incubation in the NaviCyte diffusion chambers, of colon segments from germ-free (n = 8), S7 recipient (n = 9), healthy donor recipient (n= 6), frail donor recipient (n = 7), and Frail + S7 (n = 8). Each dot represents one animal. One-way ANOVA. * p <0.05, ** p<0.01.


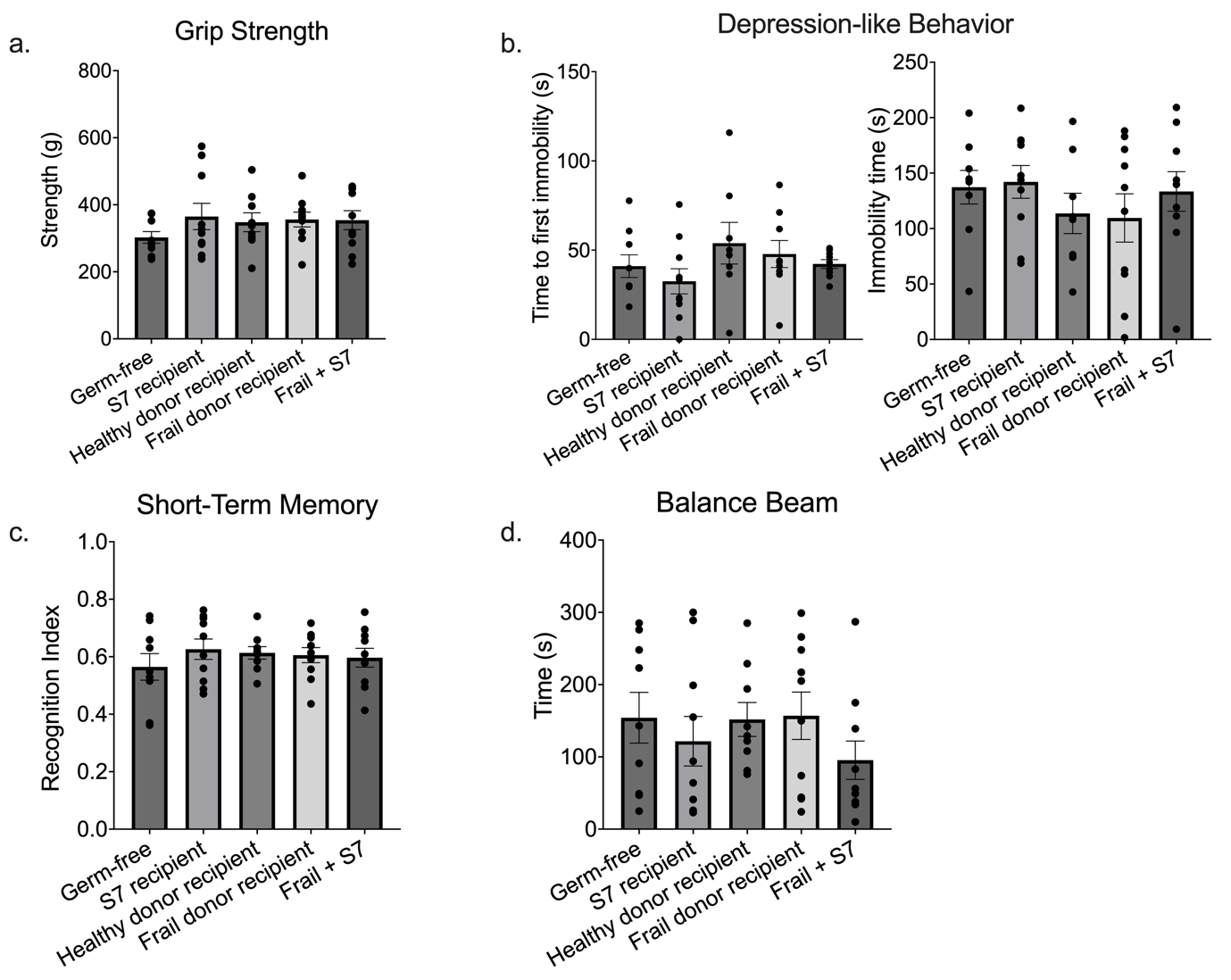


**Supplementary figure S8.** **Additional behavioral tests.** **a.** Grip strength. No significant differences were observed between groups. **b.** Depression-like behavior assessed by tail suspension test. Neither FMT nor S7 supplementation affected the time to first immobility or the total immobility time. **c.** Short-term memory in the Novel Object Recognition (NOR) test. There was no difference in short-term memory between groups. **d.** No time differences in traversing the balance beam were observed between the studied groups. Each dot represents one individual. Bars represent the Mean ± SEM. n = 9 (Germ-free), n = 10 (S7 recipient), n = 9 (Healthy donor recipient), n = 10 (Frail donor recipient), n = 10 (Frail + S7) for all figures except **b**, where n = 8 (Healthy donor recipient) for tail suspension test.


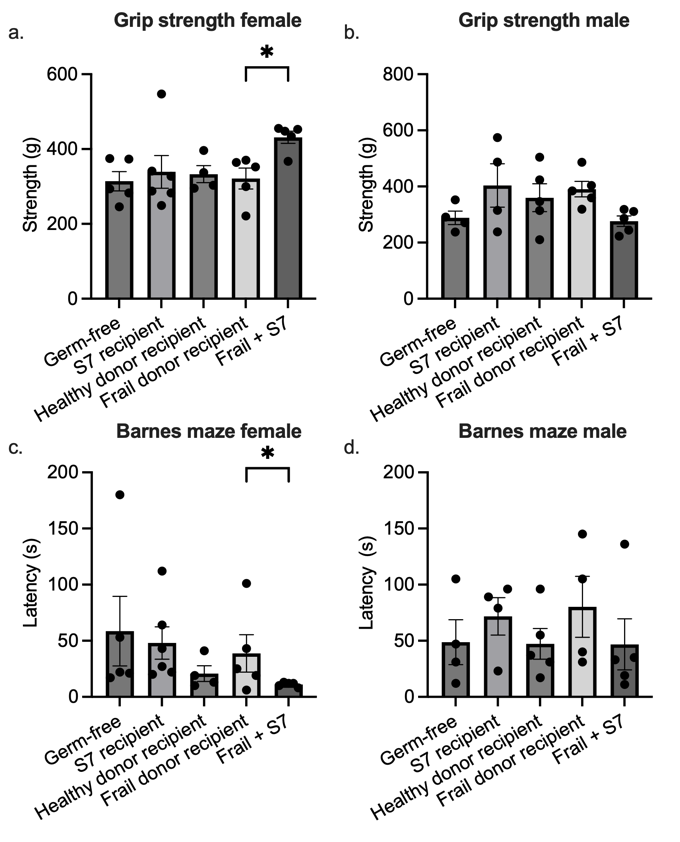


**Supplementary figure S9**. The effect of S7 treatment on female donor recipient mice. a. grip strength of female mice b. grip strength of male mice c. latency of female mice on the test day d. latency of male mice on the test day. Kruskal-Wallis post hoc Dunn’s test, * p <0.05. Germ-free (female, n = 5; male = 4), S7 recipient (female, n = 6; male, n= 4), healthy donor recipient (female, n = 4; male, n = 5), frail donor recipient (female, n = 5; male, n = 5).


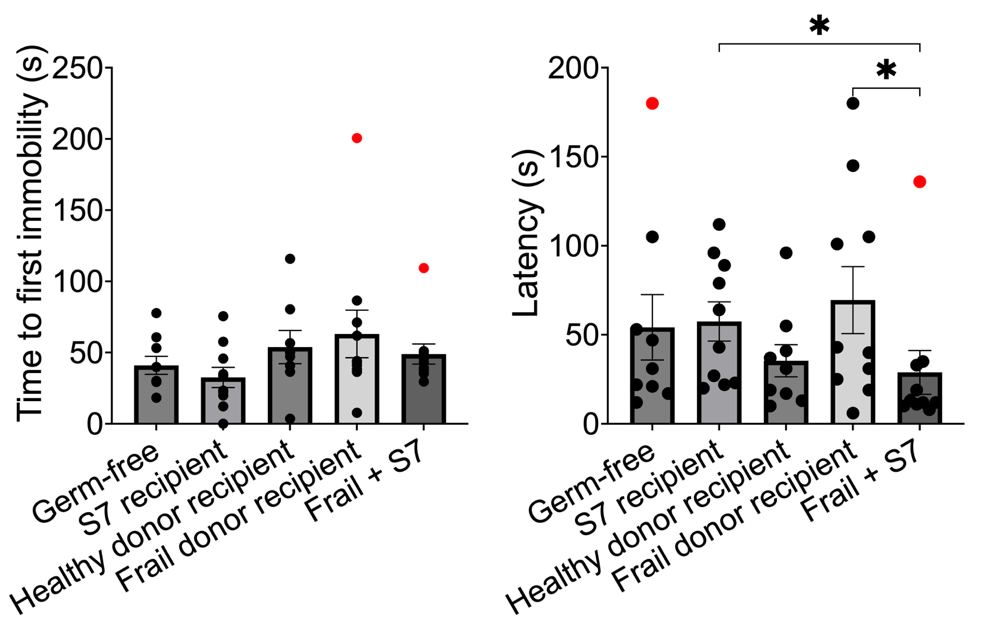


**Supplementary figure S10**. Behavior data with outliers. Left, time to first immobility in tail suspension test, n = 9 (Germ-free), n = 10 (S7 recipient), n = 8 (Healthy donor recipient), n = 9 (Frail donor recipient), n = 9 (Frail + S7); Right, Barnes maze test with outliers, n = 9 (Germ-free), n = 10 (S7 recipient), n = 9 (Healthy donor recipient), n = 10 (Frail donor recipient), n = 10 (Frail + S7). Detected outliers are marked in red. Mean±SME.

**Supplementary result**

**A significant number of unexpected taxa colonized GF recipients**

We detected the phylum Cyanobacteria in frail donor recipient mice that were not detectable in the frail donor but were present in the frail donor recipient mice 1 and 5 weeks after the transplantation (Supplementary Table S3). At weeks one and five, 13 and 14 genera, which were not detectable in the inoculum, were observed in frail donor recipient mice, including *Coprobacillus* presented with relatively high abundance (>2%) (Supplementary Table S4). In contrast to frail donor recipient mice, healthy donor recipient mice do not harbor unexpected phylum. However, twelve unexpected genera were present in healthy recipient mice at weeks one and five. Unexpected genera in healthy donor recipient mice were present with low abundance (<1%) (Supplementary Table S3 and S4). Those unexpected taxa could be taxa present in the donor microbiota below detection limits, as well as taxa originating from the environment that contaminated the GF recipient.

**Colonization of frailty-positive-related taxa in recipient mice**

A list of frailty-related genus-level taxa has been reported to elevate in Long-stay subjects compared to Community-dwelling subjects ^3^. We assessed the colonization of those frailty-positive-related taxa. In total 14 frailty-positive-related taxa, only seven and nine were detected in the healthy and frail donors, respectively. We observed an inefficient fecal bacterial transplantation of *Escherichia-Shigella* and *Streptococcus,* two of the most abundant frailty-positive-related taxa in the frail donor, from the frail donor (>10%) to its recipient mice (<1%) (Supplementary Table S11). Seven frailty-positive-related taxa exhibited higher relative abundance in frail donor recipient mice compared to healthy donor recipient mice at weeks one and five, including *Coprobacillus*, *Anaerotruncus*, *Anaerofilum*, *Eubacterium*, *Parabacteroides*, *Escherichia-Shigella,* and *Streptococcus.*
