# Supplementary figures and images for: Engraftment of aging-related human gut microbiota and the effect of a seven-species consortium in a pre-clinical model
Source: Gut Microbes. 2023 Nov 27;15(2):2282796. doi: 10.1080/19490976.2023.2282796 (PMC10854441; doi:10.1080/19490976.2023.2282796)

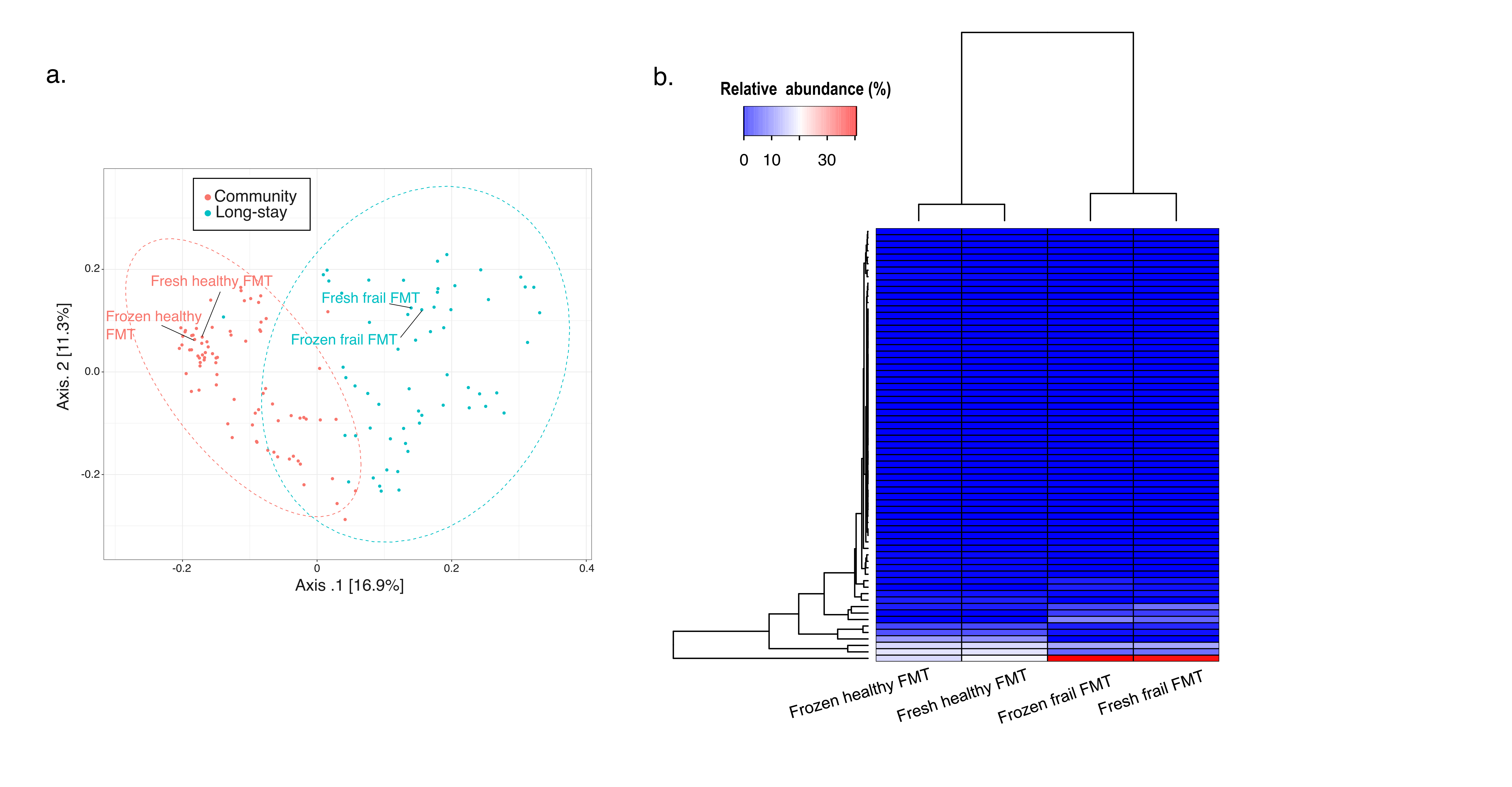

Supplement: Supplemental Material [file KGMI_A_2282796_SM5953.zip › Supplemental materials/Supplementary_figure_S1.png]

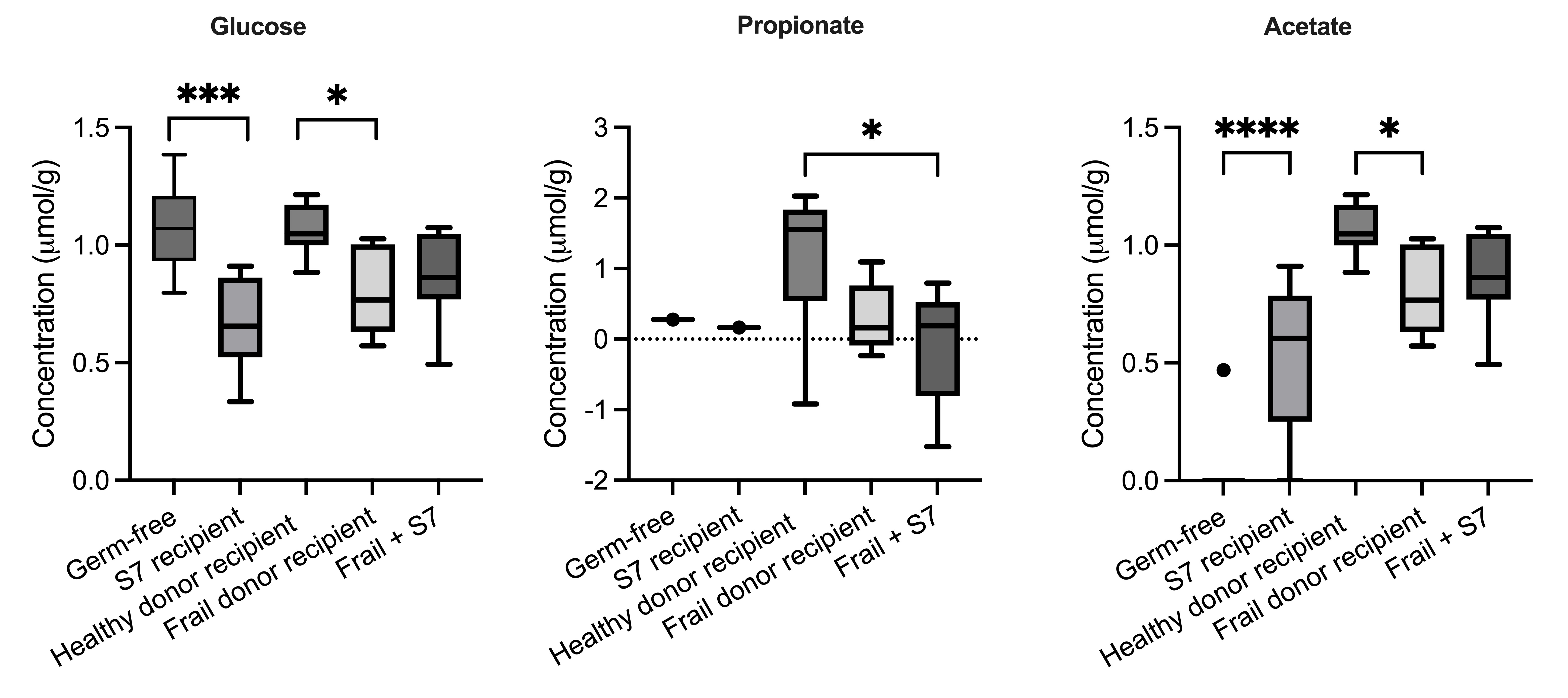

Supplement: Supplemental Material [file KGMI_A_2282796_SM5953.zip › Supplemental materials/Supplementary_figure_S5.jpg]
